# Associations between DNA Damage and PD-L1 Expression in Ovarian Cancer, a Potential Biomarker for Clinical Response

**DOI:** 10.3390/biology10050385

**Published:** 2021-04-29

**Authors:** Elise K. Mann, Kevin J. Lee, Dongquan Chen, Luciana Madeira da Silva, Valeria L. Dal Zotto, Jennifer Scalici, Natalie R. Gassman

**Affiliations:** 1 Department of Physiology and Cell Biology, College of Medicine, University of South Alabama, Mobile, AL 36688, USA; ekm1821@jagmail.southalabama.edu (E.K.M.); kjlee@health.southalabama.edu (K.J.L.); 2 Mitchell Cancer Institute, University of South Alabama, Mobile, AL 36604, USA; lsilva@health.southalabama.edu (L.M.d.S.); jscalici@health.southalabama.edu (J.S.); 3 Division of Preventive Medicine, University of Alabama at Birmingham, Birmingham, AL 35294, USA; dchen@uabmc.edu; 4 Department of Pathology, College of Medicine, University of South Alabama, Mobile, AL 36688, USA; vdalzotto@health.southalabama.edu

**Keywords:** DNA damage, ovarian cancer, PD-L1 inhibitors, immunotherapy

## Abstract

**Simple Summary:**

This work establishes that ovarian tumors contain persistent oxidative DNA damage levels that can be measured using Repair Assisted Damage Detection (RADD). The persistent DNA damage correlates with increased protein expression of PD-L1, establishing a link between genomic instability and PD-L1 expression across ovarian tumors. DNA damage may be a potential biomarker for immunotherapy.

**Abstract:**

Programmed death ligand-1 (PD-L1) inhibitors are currently under investigation as a potential treatment option for ovarian cancer. Although this therapy has shown promise, its efficacy is highly variable among patients. Evidence suggests that genomic instability influences the expression of PD-L1, but little is known about this relationship in ovarian cancer. To examine the relationship between PD-L1 expression and genomic instability, we measured DNA damage using Repair Assisted Damage Detection (RADD). We then correlated the presence of persistent DNA damage in the ovarian tumor with protein expression of PD-L1 using immunohistochemistry. Ovarian tumors showed a high prevalence of oxidative DNA damage. As the level of oxidative DNA damage increased, we saw a significant correlation with PD-L1 expression. The highest correlation between DNA damage and PD-L1 expression was observed for mucinous ovarian tumors (r = 0.82), but a strong correlation was also observed for high grade serous and endometrioid tumors (r = 0.67 and 0.69, respectively). These findings link genomic instability to PD-L1 protein expression in ovarian cancer and suggest that persistent DNA damage can be used as a potential biomarker for patient selection for immunotherapy treatment.

## 1. Introduction

Ovarian cancer is a highly complex disease with poor treatment outcomes. Although most patients respond to initial treatment with cytoreduction and combination chemotherapy, over 80% of ovarian cancers recur within the first 24 months [1]. High rates of recurrence have been attributed to delays in diagnosis. Despite 5-year survival rates of localized ovarian cancer being 92.6%, only 15.7% are diagnosed in stage 1 when the cancer is confined to the local tissue [2]. Currently, no tumor markers exist for ovarian cancer, which makes early diagnosis difficult. The promising cancer antigen CA-125 lacks sensitivity and specificity and is no longer recommended as a screening tool [3]. Thus, ovarian cancer is typically advanced when treatment is initiated.

Considering that the advanced stage contributes to disease recurrence, there is a need to improve first-line therapies to treat advanced ovarian cancers more effectively. Current guidelines for patients with late-stage ovarian cancer are complete surgical resection followed by systemic chemotherapy. Treatment with two agents is preferred and includes the combination of a platinum-based agent and a taxane. Current practice also suggests testing the tumor for somatic mutations during this initial phase of therapy [4].

It is becoming increasingly appreciated that the presence of somatic mutations in ovarian tumors influences chemotherapeutic success [4,5]. Of particular interest are mutations in DNA damage response and repair machinery proteins, such as *BRCA1/2* and *TP53*, which are associated with an increased risk of recurrence. Defects in DNA repair are associated with ~50% of ovarian cancers, and these defects cannot be solely accounted for by *BRCA* or *TP53* mutations. Current data suggest that impaired DNA damage response and repair are critical in ovarian cancer progression even though the role specific genes play is unknown [6]. Targeting DNA repair defects with poly(ADP-ribose) polymerase inhibitors (PARPis) and other DNA damage response inhibitors has shown some success in ovarian cancers [6,7]. Yet, overall responses are mixed and not conclusively linked to any mutation or gene signature.

As a result, there has been renewed interest in using immunotherapy in ovarian cancer due to the presence of tumor-infiltrating lymphocytes associated with high-grade serous, clear cell, and endometrioid histology [8]. Immunotherapy agents, including programmed death-1/programmed death-ligand 1 (PD-1/PD-L1) inhibitors, have gained popularity as a strategy for improving cancer treatment. PD-1/PD-L1 inhibitor immunotherapies inhibit immune cell tolerance. PD-1 is an inhibitory receptor expressed on activated CD4+ and CD8+ T-cells found in the peripheral tissue [9]. The receptor interacts with the ligand PD-L1 expressed on the surface of malignant cells. The interaction of PD-1 and PD-L1 leads to tolerance by the immune system, allowing cancer cells to evade destruction [10]. The immunotherapy agent nivolumab, which inhibits the PD-1/PD-L1 interaction, has recently proven effective in treating non-small cell lung cancer (NSCLC), reducing the risk of death compared to standard chemotherapy regimens [11]. Given its success, PD-1/PD-L1 inhibition is quickly becoming a promising treatment approach for cancers beyond NSCLC [12].

PD-1/PD-L1 inhibitors are currently under investigation for use in ovarian cancer in multiple clinical trials [8,13,14,15]. Evidence suggests that PD-1/PD-L1 blockade success in ovarian cancer differs based on histological pathology [15,16,17,18,19]. Although these therapies show promise in some patients, responses are highly variable among ovarian tumors, and the cause of this variability is currently unknown [14]. The lack of a biomarker to predict PD-1/PD-L1 success limits patient stratification in these clinical trial patients, further lowering the efficacy rate. There is a need for better predictive markers of PD-L1 use. PD-L1 upregulation and protein expression is one possible biomarker, but it has been ineffective for patient stratification alone to date. Additional biomarkers that coincide with PD-L1 upregulation in ovarian cancer cells could improve patient stratification and targeted application of PD-L1 blockade in ovarian cancer.

Emerging evidence suggests that defects in DNA repair machinery influence the upregulation of PD-L1 on cancer cells [20]. However, genomic signatures that characterize DNA repair defects in ovarian cancer have not been identified. Current genomic instability measurements, such as somatic mutations or gene expression changes, rely on imprecise measurements of defective DNA repair and have not identified DNA repair defects consistently. These methods are also indirect and do not measure DNA damage loads or repair capacity in tumors. In addition to somatic mutations, DNA repair defects leave persistent DNA damage within the genome [21,22,23].

DNA damage is induced by a variety of exogenous and endogenous agents. Accumulation of DNA damage has been implicated in many disease processes, including cancer, aging, and neurodegeneration [24]. Even under physiologically “normal” circumstances, a level of DNA damage exists within the cell that requires repair [21]. Estimates of the level or amount of basal DNA damage within the cell are highly variable [21,25]. However, it has been postulated that as this persistent DNA damage level increases due to aging, environmental exposures, or somatic mutations, it contributes to transformation and carcinogenesis [25,26]. Changes in DNA damage response and DNA repair pathways within cells further contribute to the persistence of DNA damage within the genome. This phenomenon has been observed when base excision repair capacity changes or in the presence of inflammatory cytokines [23,27,28].

In ovarian cancer, defects in homologous recombination coupled with high levels of oxidative stress and mitochondrial dysfunction elevate DNA damaging events and contribute to the somatic mutations and chemoresistance observed in these cancers [4,6,29]. Several studies have noted changes in DNA damage levels when defects in DNA repair pathway proteins occur, though most of these studies examine changes in DNA damage after exposure to chemotherapeutic agents [6,29,30,31]. While DNA damage response and repair is increasingly appreciated in ovarian cancer, the relationship between basal levels of DNA damage, defective DNA repair machinery, cancer development, progression, and treatment is still being investigated [6,30,32]. The known mutations in *TP53*, *BRCA1*, *PI3K*, and *KRAS* all suggest increased stress and DNA damage within ovarian tumors, but the lack of tools to measure and quantify different types of DNA lesions within tissues and tumors has hampered our understanding of the influence of unrepaired, persistent DNA damage has on tumor development, progress, and therapeutic response.

To address this limitation, we have developed a novel technique to measure DNA damage within cells and formalin-fixed paraffin-embedded (FFPE) tissues termed the Repair Assisted Damage Detection (RADD) [33,34]. RADD can measure genomic instability more precisely by measuring the persistent DNA lesions and strand breaks that remain in the genome [34,35]. RADD detects, excises, and tags DNA lesions and strand breaks within the genome using a cocktail of repair enzymes, allowing measurement of DNA repair defects within the tumor sample. With RADD, persistent DNA damage, such as abasic sites, deamination events, oxidative lesions, crosslinks, and strand breaks, can be measured within an ovarian tumor to identify DNA repair deficiencies within the tumor. DNA damage measurements offer a more accurate assessment of genomic instability within the tumor by measuring the unrepaired lesions within the genome. We hypothesize that DNA damage measurements will correlate with the expression of PD-L1. Currently, genomic instability is inferred from somatic mutations and gene expression signatures which only show a weak correlation with PD-L1 expression [20].

Here, we demonstrate the DNA damage can be measured within ovarian tumors using RADD. We also demonstrate that differences in DNA lesion content can be observed between pathological subtypes. Finally, we validate our hypothesis by showing that DNA damage levels correlate with PD-L1 expression, suggesting DNA lesions offer a better biomarker for PD-L1 expression.

## 2. Materials and Methods

### 2.1. Tumor Microarray (TMA) Samples

Three ovarian tumor microarrays were purchased from US BioMax, Inc. (Rockville, MD, USA): BC110118, OV1501b, and OV1921. The details for these microarrays can be found in Supplementary Tables S1–S3. Each array contained various ovarian tumors and normal ovary tissues from a wide age range of donors. All cores in the BC110118 and OV1501b were present for staining and analysis. In the OV1921 microarray, cores in position A3, A11, D14, G12, I7, and J14 were not present in the samples provided and were not used in the analysis. LC04, an NSCLC tissue microarray with four cores that have known PD-L1 staining, was also used.

### 2.2. Repair Assisted Damage Detection (RADD)

TMA slides were placed on a heat block set for 65 °C and incubated for 8 min to melt the paraffin. Slides were then placed directly in 100% xylene and incubated twice for 10 min each. Slides were rehydrated in water through sequential incubations in ethanol and water mixtures. Specifically, slides were incubated for 5 min each in sequential order of 100% ethanol-0% water; 70% ethanol-30% water; 50% ethanol-50% water; 30% ethanol-70% water; 0% ethanol-100% water. Rehydrated slides were then placed in glass Coplin jars with 200 mL of 10 mM sodium citrate in water and microwaved twice for 2.5 min at 120 watts until the solution reaches 47 °C for antigen retrieval. Slides were allowed to cool in water for 2 min. Slides were briefly dried, and tissue microarray was outlined with a hydrophobic barrier using a PAP pen. A lesion removal cocktail (Table 1, Lesion Processing Mix) was added to each tissue sample and incubated for 1 h at 37 °C. For the full RADD broad-spectrum lesion removal cocktail, all enzymes in Table 1 were included. For oxidative lesions only (oxRADD), AAG, T4 PDG, and UDG were omitted from the lesion removal cocktail and replaced with water (Table 2, Lesion Processing Mix). A gap-filling solution (Table 1, Gap Filling Mix) was then added directly on top of the lesion removal solution and incubated for another hour at 37 °C. Slides were washed three times in phosphate-buffered saline (PBS) for 5 min each and blocked in 2% BSA in PBS for 30 min at room temperature (RT ~ 23 °C). Anti-Digoxigenin (Dig) antibody (abcam, Cambridge, MA, USA; #ab420 clone 21H8) was incubated at a dilution of 1:250 in 2% BSA in PBS at 4 °C overnight. As a negative control for the Dig antibody, an extra slide processed with the full RADD enzyme cocktail was incubated with mouse IgG isotype control antibody (Cell Signaling, Boston, MA, USA; 5415, clone G3A1) at a dilution of 1:625 at 4 °C overnight. This dilution factor matched the µg of anti-Dig antibody used per 100 µL. The next day slides were washed three times in PBS for 5 min each, and Alexa Fluor 546 goat anti-mouse secondary (Invitrogen, Carlsbad, CA, USA; A-11003) was incubated at a dilution of 1:400 in 2% BSA in PBS for 1 h at R.T. Hoechst 33342 (Life technologies H3570) was added at a final dilution of 1:1000 for 15 min at RT to stain the nuclei. Slides were washed three times in PBS for 5 min each, briefly dried, and mounted with coverslips using ProLong Gold Antifade reagent (Life technologies P36930). Slides were allowed to dry overnight in the dark at RT and visualized using a Nikon A1R confocal microscope or stored at 4 °C until analysis.

### 2.3. PD-L1 Staining

PD-L1 was stained in parallel with oxRADD. The oxRADD protocol was completed as described above. After the 1 h incubation with the gap filling mixture, the slides were washed three times in PBS and blocked in 5% normal goat serum (Thermo Fisher 31873) in PBS for 30 min. Anti-Digoxigenin (Dig) antibody (abcam; #ab420 clone 21H8) at 1:250 and anti-PD-L1 antibody (abcam #ab205921 clone 28−8) at 1:500 was incubated in 5% normal goat serum in PBS at 4 °C overnight. The next day slides were washed three times in PBS for 5 min each, and the Alexa Fluor 546 goat anti-mouse secondary and Alexa Fluor 647 goat anti-rabbit secondary (Invitrogen) were incubated at a dilution of 1:400 in 2% BSA in PBS for 1 h at RT. Hoechst 33342 was added at a final dilution of 1:1000 for 15 min at RT to stain the nuclei. Slides were washed three times in PBS for 5 min each, briefly dried, and mounted with coverslips using ProLong Gold Antifade reagent. Slides were allowed to dry overnight in the dark at RT and visualized using a Nikon A1R confocal microscope or stored at 4 °C until analysis.

### 2.4. Image Acquisition

All images were acquired using a Nikon A1r scanning confocal microscope with a Plan-Apochromat 10×/0.5 objective. Image acquisition settings were obtained by imaging each tissue core within the TMA. Gain settings for Alexa546 and Alexa647 were determined by examining the TMAs and selecting settings that limited the number of saturated pixels. We used Alexa546 settings similar to our previous work with individual slides of ovarian tumors [33]. We established the Alexa647 PD-L1 imaging conditions using the LC04 TMA. These imaging conditions were used for all tissue imaging allowing for direct comparisons and analysis between each core. For each TMA row, cores were individually isolated and mapped. Post mapping, images were then acquired using the 10× objective for each row. The tool maps the X-Y-Z positions of individual images within the tissue slice, which are then acquired individually at 10×, 1024 × 1024 resolution for further analysis. Images of the TMAs are provided in Supplemental Figures S1–S9.

### 2.5. Image Analysis

The Nikon Elements software was used to create a binary mask of the RADD signal intensity and the PD-L1 signal intensity. The sum fluorescence intensity was exported for each channel. Gating for the binary mask (750−3500) was defined by the lowest intensity image, and these settings were used between all images for analysis for RADD, oxRADD, and PD-L1. The fluorescent intensity for each core was recorded. Data were plotted using GraphPad Prism software.

### 2.6. Statistical Analysis

The results are reported as mean ± the standard error of the mean (SEM) with the n for each group indicated. An unpaired two-tailed *t*-test analyzed differences between the two groups. Differences between more than two groups were analyzed by one-way analysis of variance (ANOVA) with a Dunnett’s post hoc test. Pearson’s correlation was analyzed in GraphPad Prism with a two-tailed test used for significance.

## 3. Results

### 3.1. Ovarian Tumors Have Persistent DNA Damage

We previously measured DNA damage in a small cohort of ovarian cancer patients [33]. Samples from these patients included matched pairs of de-identified ovarian tumors pre-and post-treatment with carboplatin and paclitaxel. Using RADD, we measured differences in the basal levels of DNA damage among the ovarian tumor samples. Clear differences were observed between the samples, but only a single pathological type was measured.

To extend our original study, we purchased three different tumor microarrays (TMAs) that contained various ovarian tumors and normal tissues. OV1921 included late-stage ovarian tumors consisting of 192 cores in mid to advanced stage. OV1501b consisted of 150 cores representing a variety of ovarian cancer types. BC110118 consisted of 72 cores of various ovarian cancer types. Details regarding each core in the TMAs can be found in Supplemental Tables S1–S3.

**Figure 1:**
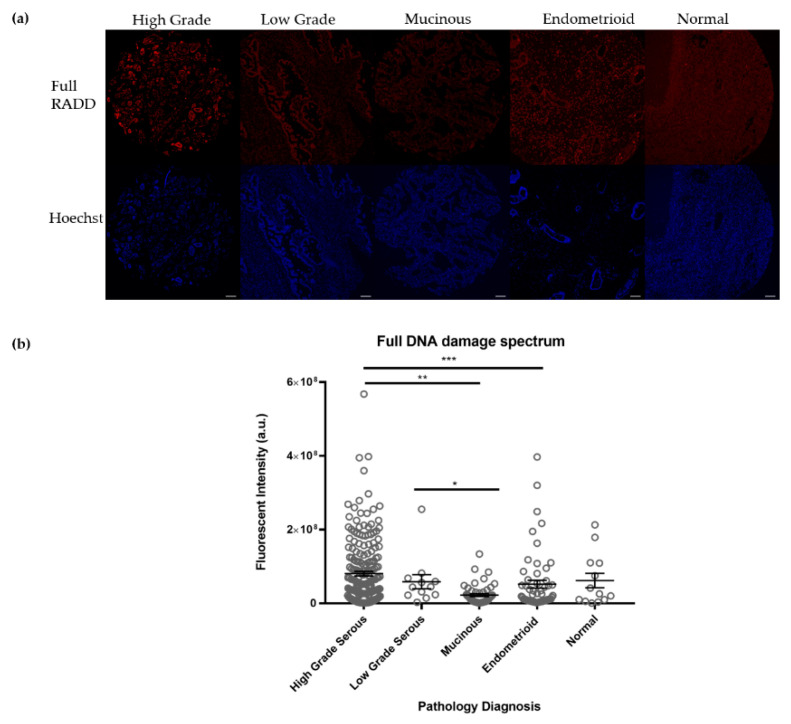
Broad spectrum DNA damage levels across pathological subtypes. (**a**) Representative images of tumor cores from each pathological subtype. Scale bar = 100 µm. (**b**) Quantification of the full broad spectrum DNA damage level (Full RADD) within the major pathological subtypes contained on the TMAs. Full data can be found in Supplemental Information. *** *p* < 0.001, ** *p* < 0.01, and * *p* < 0.05.

We conducted a broad spectrum analysis of the persistent DNA damage contained within the 408 samples using the Full RADD cocktail described in Table 1. This cocktail detects abasic sites, alkylated damage, deamination events, DNA crosslinks, oxidative DNA damage, and strand breaks [33,34]. Using this cocktail, we measured DNA damage levels within the tumor types on the TMA (Figure 1). High grade serous tumors, which contain p53 mutations, showed the highest level of DNA damage, 8.1 ± 0.6 × 10^7^ (mean ± standard error of the mean (SEM), *n* = 206). Endometrioid tumors showed a DNA damage level of 5.2 ± 1.1 × 10^7^ (*n* = 58). Mucinous tumors showed a DNA damage level of 2.2 ± 0.39 × 10^7^ (*n* = 49). Normal ovarian tissue showed a DNA damage level of 6.2 ± 2.0 × 10^7^ (*n* = 13). Low grade serous tumors showed a DNA damage level of 5.9 ± 1.9 × 10^7^ (*n* = 12). There were significant differences in DNA damage levels observed between high grade serous and mucinous (*p* < 0.001), high grade serous and endometrioid (*p* < 0.01), and low grade serous and mucinous (*p* < 0.05), but no significant differences in DNA damage levels were noted between the tumor types and the normal tissue.

### 3.2. Oxidative Lesions Are the Predominant Lesions Observed in Ovarian Cancer

**Figure 2:**
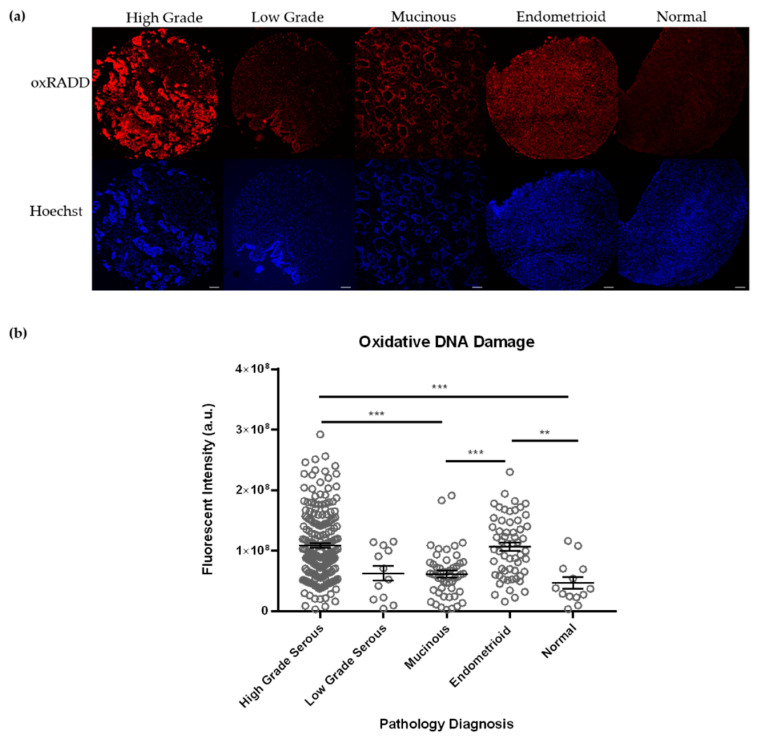
Oxidative DNA damage levels across pathological subtypes. (**a**) Representative images of tumor cores from each pathological subtype. Scale bar = 100 µm. (**b**) Quantification of the oxidative DNA damage levels (oxRADD) within the major pathological subtypes contained on the TMAs. Full data can be found in Supplemental Information. *** *p* < 0.001 and ** *p* < 0.01.

**Figure 3:**
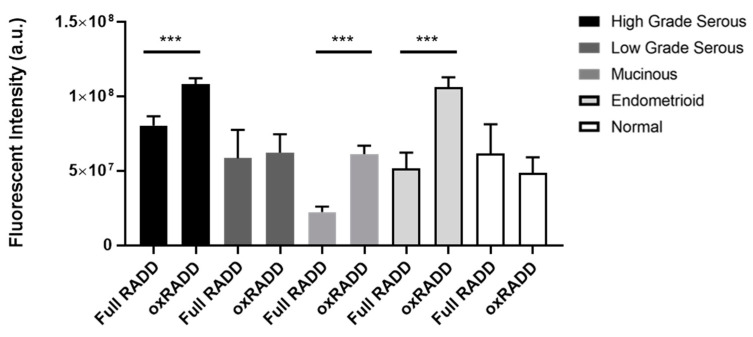
Comparing the Full RADD cocktail to an oxidative DNA damage-specific cocktail (oxRADD) across the pathological subtypes. The mean fluorescent intensity with the standard error of the mean (SEM) is shown in the bar graph for full RADD and oxRADD for each subtype. *** *p* < 0.001.

We have previously used individual lesion class cocktails to dissect the predominant lesions in a tumor sample [33]. Ovarian tissues undergo cycles of oxidative stress during ovulation, so we applied a cocktail specific to oxidative lesions (Fpg, EndoIV, and EndoVIII, termed oxRADD) to specifically examine the content of oxidative lesion with the tumor microarray samples (Figure 2). Interestingly, we noted much higher levels of DNA damage with the oxRADD cocktail for the high grade serous (1.1 ± 0.04 × 10^8^ vs. 8.1 ± 0.6 × 10^7^, *p* < 0.001), mucinous (6.1 ± 0.6 × 10^7^ vs. 2.2 ± 0.4 × 10^7^ *p* < 0.0001), and endometrioid (1.1 ± 0.06 × 10^8^ vs. 5.2 ± 1.1 × 10^7^ *p* < 0.0001) when compared to the full RADD (Figure 3). Though neither the normal tissues nor the low grade serous showed a significant difference between the two DNA damage cocktails. The oxRADD analysis also showed significant differences in DNA damage levels for high grade serous and endometrioid compared to the normal tissues (Figure 2).

Given the number of enzymes used in the broad spectrum cocktail (Full RADD), we hypothesize that the DNA becomes too fragmented to label with the Klenow, resulting in reduced overall fluorescence intensity. The reduced number of enzymes in the oxRADD cocktail leaves less fragmented DNA, allowing better tagging and a higher fluorescence intensity. We predict the full damage spectrum is more elevated than measured by full RADD due to this inefficient labeling of fragmented DNA. Since we had a limited number of tumor microarray slides for analysis, further cocktail testing could not be completed. However, future studies should test multiple cocktails to characterize the potential lesion content of the tumors fully. We will use the oxRADD for further analysis since it is a significant DNA damage component for these tumors and show a clear difference between the normal tissues (Figure 3).

### 3.3. PD-L1 Expression Correlates with DNA Damage Content

**Figure 4:**
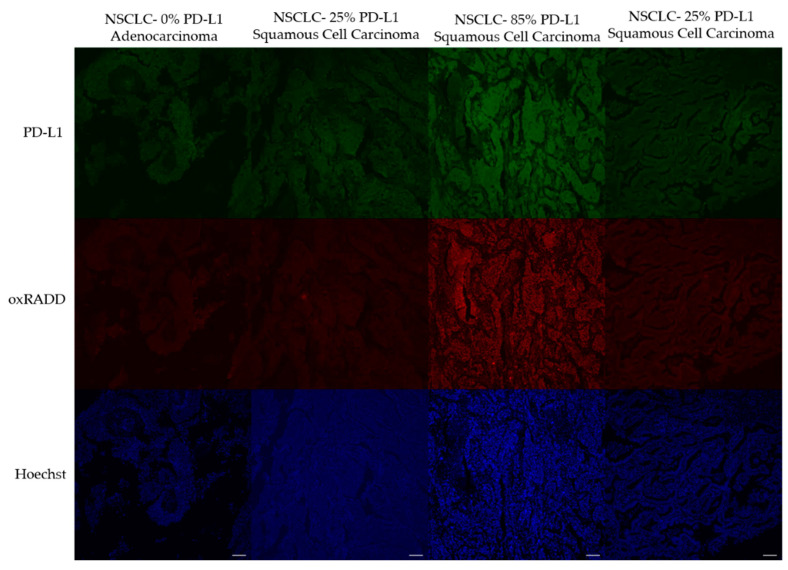
oxRADD and PD-L1 intensities in NSCLC tissues with known PD-L1 concentrations. Tumor core images from the control LC04 microarray. Scale bar = 100 µm.

To examine the relationship between oxidative DNA damage levels and PD-L1 expression, we first had to optimize the PD-L1 staining. We purchased a TMA with characterized levels of PD-L1 expression to validate our staining procedures. The LC04 TMA contains NSCLC cores with known concentrations of PD-L1 expression (25%, 85%, 25%, 0%). We first attempted to immunodetect PD-L1 using a standard immunohistochemistry protocol, and the image analysis showed unspecific staining of PD-L1 when using this method [36]. Various antigen retrieval and blocking strategies were tested consistent with existing literature, but staining was still not representative of the 0%, 25%, 85%, 25% [37,38]. We have previously multiplexed antibody staining with the RADD method [33], so we finally tested staining PD-L1 in parallel with oxRADD with the modification of using 5% goat serum as a blocking buffer rather than 2% BSA. When we stained for PD-L1 after the RADD enzyme cocktail, we saw specific staining that reflected the expected 0%, 25%, 85%, 25% PD-L1 content (Figure 4).

**Figure 5:**
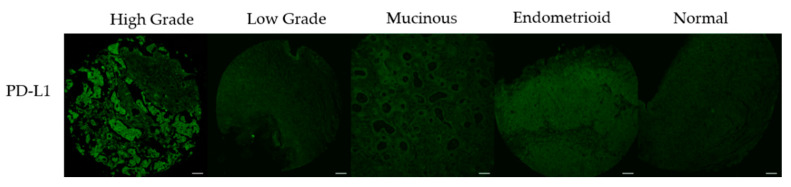
PD-L1 images across the pathological subtypes from the TMAs. Representative images of tumor cores matched to the oxRADD from each pathological subtype. Scale bar = 100 µm.

**Figure 6:**
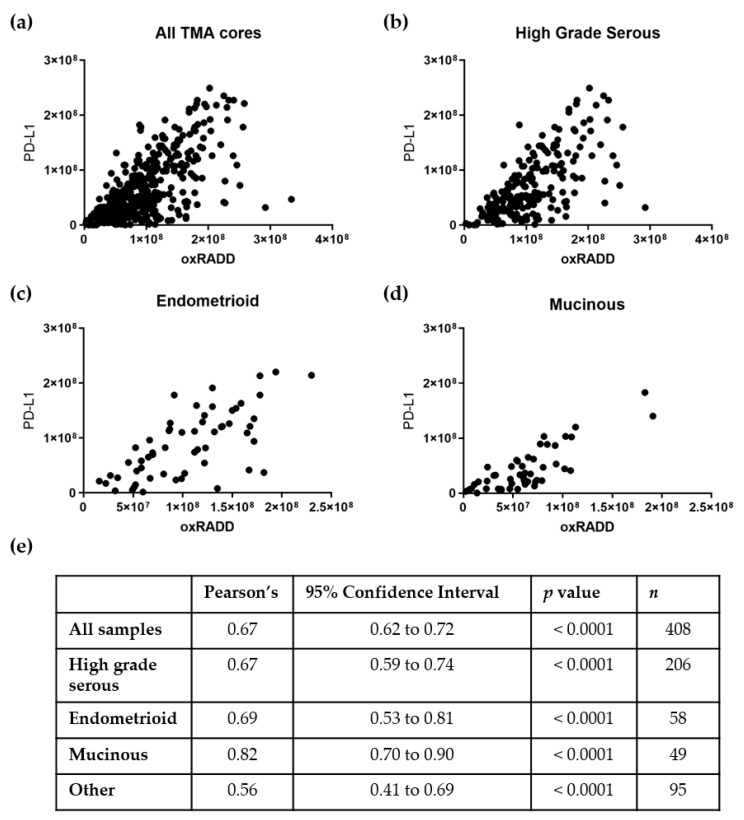
Pearson’s correlation of PD-L1 expression and oxidative DNA damage for all ovarian TMAs (**a**) and individual pathological subtypes (**b**–**d**). (**e**) Table of summary statistics for the correlation analysis.

With the PD-L1 staining now consistent with the control slide, we analyzed PD-L1 expression across the TMAs and correlated the protein expression to the oxidative DNA damage content (oxRADD, Figure 5). Figure 6 shows the correlations of PD-L1 to oxRADD. Without any filtering for pathological subtypes, we see good correlation of PD-L1 expression with DNA damage level (r = 0.69). When we further examined the correlation within the pathological subtypes with a larger number of cores, we saw that both high grade serous and endometrioid showed a good correlation between oxRADD and PD-L1 (r = 0.67 and 0.69, respectively). Interestingly, the mucinous subtype showed the highest correlation between DNA damage and PD-L1 expression (r = 0.82).

## 4. Discussion

Immune checkpoint blockade of PD-1/PD-L1 is being widely tested against different cancer types, with various levels of success [39]. Despite the presence of immune infiltrates in patients with ovarian cancer, PD-1/PD-L1 blockade has shown limited effectiveness in clinical trials with only a modest 9–15% response rate [15,40,41]. As such, there are no FDA-approved immunotherapies for ovarian cancer. The efficacy of immune checkpoint inhibitors in other solid tumor models suggests that better biomarkers are needed for selecting patients for PD-L1 trials [18].

Immunohistochemistry analysis of PD-L1 protein expression is frequently used for patient selection, but PD-L1 expression does not always identify patients who will respond to PD-L1 blockade. Recently, several studies have identified defects in DNA damage response and repair proteins as potential predictive biomarkers for immunotherapy response [20,42,43]. However, genetic signatures and gene expression profiles are still being developed and validated. One significant challenge has been linking somatic mutations or gene expression signatures with DNA repair defects. Except for *BRCA* and *TP53*, few genes are well-correlated with DNA repair defects in ovarian and other cancers. While it has been estimated at roughly 50% of epithelial ovarian cancers contain DNA repair defects, the nature of these defects is poorly understood [32].

A better strategy for understanding the DNA repair status of ovarian tumors is to measure persistent DNA damage within the genome. Both Bartek et al. and Khanna have elegantly described potential DNA damage tolerance levels that occur in cancer cells from defective DNA repair and the accumulation of somatic mutations [25,26]. The persistence and tolerance of DNA damage within the genome further contribute to tumorigenesis and impact the tumor’s therapeutic response [25,26]. Evidence of the existence of these DNA damage thresholds has been lacking due to our inability to measure the broad spectrum of DNA damage cells contain. To address this issue, we developed the RADD method to directly measure DNA damage in isolated DNA, cell lines, and tissues to better characterize and quantify DNA repair defects [33,34,35].

RADD measures persistent DNA damage in the form of base lesions and strand breaks within the genome [33,34,44]. Here, we used commercially available ovarian TMAs with various cores reflecting normal tissue, different pathological subtypes, and mixed pathological subtypes to demonstrate the broad spectrum full RADD cocktail could detect persistent DNA damage in ovarian cancer. We observed a range of DNA damage levels across the pathological subtypes with the full RADD cocktail. High grade serous tumors showed significantly higher levels of damage compared to mucinous and endometrioid (*p* < 0.001 and *p* < 0.01, respectively; Figure 1). However, none of the pathological subtypes showed a significantly higher DNA damage level than the normal tissues. While this lack of significant difference may be due to the low number of normal tissues (*n* = 12), we decided to examine the DNA damage content using a subset of DNA repair enzymes from the full RADD cocktail.

Ovarian tissues are known to undergo cycles of oxidative stress, so we used a cocktail of oxidative DNA glycosylases (Fpg, EndoIV, and EndoVIII; oxRADD; Table 2) to measure oxidative DNA damage left within the tumor cells (Figure 2). We observed an increase in DNA damage for high grade serous, endometrioid, and mucinous subtypes (Figure 3) when using the oxRADD cocktail. Significant differences in oxidative damage content were observed between the high grade serous and endometrioid tumors compared to the normal tissues (Figure 2). These results indicated that oxidative DNA lesions are predominant lesions within the high grade serous, endometrioid, and mucinous subtypes, but other types of DNA damage, i.e., alkylation, deamination, or crosslinks, are also present and need to be quantified in future work. Interestingly, no significant difference in damage measurements was observed between full RADD and oxRADD for the low grade serous and normal tissues. These tissues showed moderate or low DNA damage levels and indicate oxidative lesions are the predominant lesion class.

This outcome highlighted an important limitation in the RADD assay when high DNA damage levels are present within a cell or tumor. The exo^-^ Klenow enzyme can synthesize double-stranded DNA from single-stranded templates and blunt and tag DNA ends. However, Klenow binding and polymerase activity is dependent on DNA structure and length [45]. If the DNA becomes too fragmented, then Klenow polymerase efficiency drops, and the labeling with the modified dUTP moiety used for detecting the DNA damage is lowered. This limitation may be overcome by reducing the concentration of enzymes or removing specific enzymes like UDG or AAG used in the full cocktail (Table 1). Still, these changes will likely underestimate DNA damage levels in the tissue.

Given the high content of oxidative lesions, we then used the oxRADD cocktail for further analysis and examined the expression of PD-L1 in the ovarian TMAs. We optimized the staining of PD-L1 using a control TMA of NSCLC and determined that the RADD protocol could be multiplexed with the PD-L1 antibody to allow co-staining of oxidative DNA damage and PD-L1 expression (Figure 4 and Figure 5). We then used a Pearson’s correlation to examine the relationship of PD-L1 staining to oxidative DNA content in the ovarian tumor samples. Without any filtering, we observed good correlation of the PD-L1 staining with oxRADD (Figure 6). As oxidative DNA damage content increased, so did the expression of PD-L1 in the tumors.

With the specific subtypes, mucinous tumors showed the highest correlation between oxidative DNA damage and PD-L1 expression (r = 0.82, Figure 6d). Mucinous ovarian tumors are clinically and morphologically distinct from the other subtypes of ovarian cancer. Mucinous tumors are not often associated with *BRCA* or *TP53* mutations and often contain *KRAS* mutations, leading to increased levels of oxidative stress [46]. These tumors also have a poorer response to conventional chemotherapy [47]. Mucinous ovarian tumors are rare, so specific responses of this class of tumors to PD-L1 blockade are still developing, but the high expression of PD-L1 and known increase in TILs make it an interesting target for immunotherapy [17,19]. High grade serous tumors also showed good correlation (r = 0.67) with PD-L1 expression. Unlike mucinous tumors, high grade serous tumors typically do not express *BRAF* and *KRAS* mutations. These tumors more commonly express mutations in *TP53* and *BRCA1/2* [48]. Since DNA repair defects are still highly associated with this subtype, we expected a higher correlation within this group [6]. One confounding factor may be the presence of both inactivating and activating mutations in p53 in these tumors [6]. A secondary stratification by p53 mutation status may improve the correlation in this category, but this information was not available. We also observed a good correlation between PD-L1 expression and endometrioid tumors. Endometrioid tumors typically have mutations in the mismatch repair pathways, which result in microsatellite instability [8]. Other pathological subtypes were present on the TMAs, but the case numbers were insufficient to allow correlation analysis (Supplemental Table S1).

We hypothesized that DNA damage could be measured within ovarian tumors and correlated with PD-L1 expression. The work here demonstrates that ovarian tumors contain levels of unrepaired, persistent DNA damage that can be quantified by RADD. Comparisons with DNA damage content from other tumors are not possible because DNA damage levels are not directly measured for ovarian tumors because of a lack of methodologies to accomplish this task. Genomic instability is more frequently inferred based on tumor mutational burdens and gene expression signatures. Our results are consistent with the genomic instability predicted for high grade serous and other ovarian tumor subtypes [6,30]. The RADD assay also establishes that oxidative DNA lesions are a highly prevalent lesion type in major epithelial ovarian cancer subtypes and correlates well with PD-L1 expression. Previous studies have suggested that oxidative DNA damage and antioxidant response influences ovarian tumor response to chemotherapy [29,31]. Further work with oxRADD could likely help define the mechanisms by which changes in oxidative DNA damage repair and antioxidant systems contribute to chemotherapeutic resistance.

Finally, we have demonstrated for the first time that increasing levels of DNA damage within the tumor correlates with PD-L1 expression, validating our hypothesis. Numerous studies have demonstrated that PD-L1 expression is regulated by genomic alterations such as amplifications or translocations, epigenetic modifications, and transcriptional regulation by inflammatory cytokines and oncogenic signals (as reviewed in [49]). Inflammatory signals from interleukins and oncogenic signals from proteins like MYC and EGFR are linked to increased reactive oxygen species within the cell [50,51,52]. These increased DNA damage levels alter DNA damage signaling and contribute to the persistence of DNA damage, which is directly measured by oxRADD. Therefore, oxRADD offers a unique biomarker for changes in PD-L1 expression that encompasses the many mechanisms that drive PD-L1 transcription.

The main limitation of this analysis is that DNA damage levels cannot be associated with immunotherapy response since commercially available TMAs were used to measure both PD-L1 and DNA damage. To further validate the use of DNA damage as a potential biomarker for immunotherapy response, oxRADD should be used in pre-treatment samples and DNA damage levels correlated with recurrent free survival or overall survival. Despite this limitation, the work here does establish that genomic instability in the form of persistent DNA damage is related to PD-L1 expression [49].

## 5. Conclusions

Persistent DNA damage can be measured within FFPE samples using RADD and allows quantification of genomic instability. Tailoring the enzyme cocktail used in RADD identified oxidative DNA damage as the dominant lesion type in ovarian tissues and tumors. Increasing oxidative DNA damage levels showed a significant correlation with the increased PD-L1 expression for high grade serous, endometrioid, and mucinous subtypes. These data suggest oxidative DNA damage alone or in conjunction with PD-L1 expression may be a potential biomarker to improve patient stratification for PD-L1 blockade.

## Figures and Tables

**Table 1:** Broad spectrum Full RADD assay cocktail.

Lesion Processing Mix	For a 100 µL Total Reaction Volume	Gap Filling Mix	For a 100 µL Total Reaction Volume
UDG (New England Biolabs (NEB), Ipswich, MA, USA; M0280)	2.5 U	Klenow exo^−^ (Thermo Fisher, Waltham, MA USA; P0422)	1.0
FPG (NEB M0240)	4 U	Digoxigenin dUTP (Sigma Aldrich 11093088910)	0.1
T4 PDG (NEB M0308)	5 U	Thermo Pol Buffer (NEB B9004)	10 µL
EndoIV (NEB M0304)	5 U		
EndoVIII (NEB M0299)	5 U		
AAG (NEB M0313)	5 U		
NAD^+^ (100×, NEB B9007)	500 µM		
BSA (Sigma Aldrich, St. Louis, MO, USA; A2058)	200 µg/mL		
Thermo Pol Buffer (NEB B9004)	10 µL		

**Table 2:** Oxidative lesion RADD (oxRADD) assay cocktail.

Lesion Processing Mix	For a 100 µL Total Reaction Volume	Gap Filling Mix	For a 100 µL Total Reaction Volume
FPG (NEB M0240)	4 U	Klenow exo^−^ (Thermo Fisher EP0422)	1.0
EndoIV (NEB M0304)	5 U	Digoxigenin dUTP (Sigma Aldrich 11093088910)	0.1
EndoVIII (NEB M0299)	5 U	Thermo Pol Buffer (NEB B9004)	10 µL
NAD^+^ (100×, NEB B9007)	500 µM		
BSA (Sigma Aldrich A2058)	200 µg/mL		
Thermo Pol Buffer (NEB B9004)	10 µL		

## Data Availability

Raw data generated is available in the supplementary materials.

## Supplementary Materials

The following are available online at https://www.mdpi.com/article/10.3390/biology10050385/s1, Figure S1: OV1921 Full RADD, Figure S2: OV1921 oxRADD, Figure S3: OV1921 PD-L1, Figure S4: OV1501b Full RADD, Figure S5: OV1501b oxRADD, Figure S6: OV1501b PD-L1, Figure S7: BC110118A1 Full RADD, Figure S8: BC110118A1 oxRADD, Figure S9: BC110118A1 PD-L1, Table S1: OV1921 TMA, Table S2: OV1501b TMA, Table S3: BC110118A1 TMA.
