# Supplementary material for: Associations between DNA Damage and PD-L1 Expression in Ovarian Cancer, a Potential Biomarker for Clinical Response
Source: Biology (Basel). 2021 Apr 29;10(5):385. doi: 10.3390/biology10050385 (PMC8146974; doi:10.3390/biology10050385)
Supplement: Supplementary file 1 [file biology-10-00385-s001.zip › Supplementary Material_paper.pdf]

Supplementary Material

Table S1. OV1921 TMA.

Position	Age	Pathology Diagnosis	TNM	Full RADD	oxRADD	PD-L1
A1	69	Serous papillary adenocarcinoma	T2bN0M0	46687645	7172893	12999527
A2	72	Serous papillary adenocarcinoma (sparse)	T2N0M0	94871183	38254279	20815921
A4	36	Serous papillary adenocarcinoma	T2aN0M0	1.07E+08	92304513	16892578
A5	57	Serous papillary adenocarcinoma	T2aN0M0	1.57E+08	1.12E+08	1.14E+08
A6	45	Serous papillary adenocarcinoma	T2aN0M0	1.45E+08	1.07E+08	1.41E+08
A7	35	Serous papillary adenocarcinoma	T2N0M0	75210124	45406843	52470239
A8	50	Mucinous papillary adenocarcinoma	T2N0M0	22989721	7942155	20915685
A9	69	Serous papillary adenocarcinoma	T2bN0M0	45927405	21494934	15868095
A10	72	Serous papillary adenocarcinoma	T2N0M0	57166073	25990061	64899298
A12	36	Serous papillary adenocarcinoma	T2aN0M0	72385108	54141069	99042239
A13	57	Serous papillary adenocarcinoma	T2aN0M0	84069175	75995077	23669151
A14	45	Serous papillary adenocarcinoma	T2aN0M0	63064697	31691999	1.64E+08
A15	35	Serous papillary adenocarcinoma	T2N0M0	36081135	14723201	27109946
A16	50	Mucinous papillary adenocarcinoma	T2N0M0	35021576	7634979	48127639
B1	62	Serous papillary adenocarcinoma	T2bN0M0	94611163	30899963	47397085
B2	60	Serous papillary adenocarcinoma	T2aN0M0	96828469	70908081	48461400
B3	49	Serous papillary adenocarcinoma	T2aN0M0	97364876	83648526	36730508
B4	49	Serous papillary adenocarcinoma	T2cN0M0	73787841	26800946	91406475
B5	73	Serous papillary adenocarcinoma	T2aN0M0	1.19E+08	57983726	77341345
B6	63	Serous papillary adenocarcinoma (sparse)	T2bN0M0	1.01E+08	1.07E+08	22842619
B7	45	Serous papillary adenocarcinoma	T2N0M0	1.31E+08	1.36E+08	26506236

B8	51	Serous papillary adenocarcinoma (sparse)	T2aN0M0	69237306	52487481	7173604
B9	62	Serous papillary adenocarcinoma (fibrofatty tissue)	T2bN0M0	1.78E+08	59526441	1.44E+08
B10	60	Serous papillary adenocarcinoma	T2aN0M0	53270639	28872068	34905725
B11	49	Serous papillary adenocarcinoma	T2aN0M0	50700658	16791951	39264385
B12	49	Serous papillary adenocarcinoma	T2cN0M0	1.13E+08	54872259	85828561
B13	73	Serous papillary adenocarcinoma	T2aN0M0	1.4E+08	68047951	72805790
B14	63	Serous papillary adenocarcinoma	T2bN0M0	1.13E+08	1.26E+08	25455100
B15	45	Serous papillary adenocarcinoma	T2N0M0	65769260	54526699	31403836
B16	51	Serous papillary adenocarcinoma	T2aN0M0	41002058	19744249	8312740
C1	49	Serous adenocarcinoma	T2N0M0	1.01E+08	9312770	14374533
C2	60	Serous papillary adenocarcinoma	T2bN0M0	67143281	27477220	10025627
C3	39	Serous adenocarcinoma (necrotic tissue)	T2N0M0	88060448	46963994	51985246
C4	56	Serous adenocarcinoma	T2N0M0	1.27E+08	97774181	1.68E+08
C5	51	Serous adenocarcinoma	T2aN0M0	77646377	83957919	46850941
C6	51	Serous adenocarcinoma	T2bN0M0	2.13E+08	2.18E+08	2.01E+08
C7	45	Serous adenocarcinoma	T2bN0M0	1.93E+08	1.86E+08	10687226
C8	51	Serous adenocarcinoma	T2N0M0	1.42E+08	1.08E+08	2.97E+08
C9	49	Serous adenocarcinoma	T2N0M0	1.65E+08	43143720	30421334
C10	60	Serous papillary adenocarcinoma	T2bN0M0	81339496	82845952	11899820
C11	39	Serous adenocarcinoma	T2N0M0	93747674	1.07E+08	21608611
C12	56	Serous adenocarcinoma	T2N0M0	1.6E+08	99209323	93976342
C13	51	Serous adenocarcinoma	T2aN0M0	1.35E+08	1.31E+08	17774306
C14	51	Serous adenocarcinoma	T2bN0M0	1.69E+08	2.11E+08	26912948
C15	45	Serous adenocarcinoma	T2bN0M0	70601133	74975563	32852768
C16	51	Serous adenocarcinoma	T2N0M0	79125918	15645936	2.35E+08
D1	43	Serous adenocarcinoma	T2N0M0	72336733	49400468	1137892
D2	56	Serous papillary adenocarcinoma	T2N0M0	1.55E+08	92917455	1.35E+08
D3	65	Serous papillary adenocarcinoma	T2N0M0	1.59E+08	59578446	1.25E+08
D4	63	Serous adenocarcinoma	T2N0M0	1.32E+08	1.02E+08	1.1E+08
D5	75	Serous adenocarcinoma	T2N0M0	1.53E+08	1.74E+08	56973210
D6	41	Serous adenocarcinoma	T2cN0M0	1.78E+08	58681090	2.25E+08

D7	56	Serous adenocarcinoma	T2N0M0	1.14E+08	59688103	78173124
D8	48	Serous adenocarcinoma	T2aN0M0	1.24E+08	55261585	1.49E+08
D9	43	Serous adenocarcinoma	T2N0M0	1.21E+08	1.44E+08	4927234
D10	56	Serous papillary adenocarcinoma	T2N0M0	2.31E+08	1.91E+08	1.54E+08
D11	65	Serous papillary adenocarcinoma	T2N0M0	2.56E+08	1.78E+08	1.31E+08
D12	63	Serous adenocarcinoma	T2N0M0	2.46E+08	1.09E+08	1.87E+08
D13	75	Serous adenocarcinoma	T2N0M0	2.4E+08	1.26E+08	1.05E+08
D15	56	Serous adenocarcinoma	T2N0M0	1.14E+08	73411595	51931308
D16	48	Serous adenocarcinoma	T2aN0M0	1.01E+08	16000376	69616850
E1	29	Mucinous papillary adenocarcinoma	T2aN0M0	62041914	36646530	2289352
E2	50	Mucinous papillary adenocarcinoma	T2N0M0	74014511	23314883	20159640
E3	41	Mucinous papillary adenocarcinoma	T2aN0M0	56917434	33435566	1091804
E4	62	Mucinous papillary adenocarcinoma	T2aN0M0	64794574	20853282	22067500
E5	50	Mucinous adenocarcinoma	T2N0M0	46740888	7985952	92445352
E6	41	Mucinous adenocarcinoma	T2aN0M0	1.13E+08	1.2E+08	5402673
E7	46	Mucinous papillary adenocarcinoma	T2N0M0	79091387	22589809	17256708
E8	60	Adenocarcinoma (necrotic tissue)	T2N0M0	2.25E+08	41706633	1.43E+08
E9	29	Mucinous papillary adenocarcinoma	T2aN0M0	67571941	34922127	7360411
E10	50	Mucinous papillary adenocarcinoma	T2N0M0	59259273	48842265	10428783
E11	41	Mucinous papillary adenocarcinoma	T2aN0M0	93341278	52906962	3475426
E12	62	Mucinous papillary adenocarcinoma	T2aN0M0	72562869	20595666	15832648
E13	50	Mucinous adenocarcinoma	T2N0M0	71657982	12800875	1.34E+08
E14	41	Mucinous adenocarcinoma	T2aN0M0	81112210	1.03E+08	1849780
E15	46	Mucinous papillary adenocarcinoma	T2N0M0	1.08E+08	40859670	9841103
E16	60	Adenocarcinoma (necrotic tissue)	T2N0M0	3.34E+08	46932211	3.68E+08
F1	50	Endometrioid adenocarcinoma	T2aN0M0	93164504	23628819	16236418
F2	68	Endometrioid adenocarcinoma	T2cN0M0	50969625	10126659	9554763
F3	29	Endometrioid adenocarcinoma	T2aN0M0	1.15E+08	78816174	24937445

F4	47	Endometrioid adenocarcinoma	T2aN0M0	66536213	96211587	1452740
F5	48	Clear cell carcinoma	T2N0M0	1.53E+08	18552856	1.7E+08
F6	47	Clear cell carcinoma	T2aN0M0	82091398	85671819	21556831
F7	37	Embryonal carcinoma	T2N0M0	83081000	43404412	2379375
F8	47	Squamous cell carcinoma from malignant transformation of teratoma	T2aN0M0	99719605	62928249	8030602
F9	50	Endometrioid adenocarcinoma	T2aN0M0	1.02E+08	35568328	47524662
F10	68	Endometrioid adenocarcinoma	T2cN0M0	80799744	34380849	9810302
F11	29	Endometrioid adenocarcinoma	T2aN0M0	52389145	14905299	37764254
F12	47	Endometrioid adenocarcinoma	T2aN0M0	34325748	27644429	3335389
F13	48	Clear cell carcinoma	T2N0M0	1.64E+08	11898301	3.48E+08
F14	47	Clear cell carcinoma	T2aN0M0	42280270	50745240	23540451
F15	37	Embryonal carcinoma	T2N0M0	51523434	60824379	3887743
F16	47	Squamous cell carcinoma from malignant transformation of teratoma	T2aN0M0	97950471	73287704	3725128
G1	41	Malignant granulosa cell tumor	T2N0M0	1.09E+08	1.04E+08	2299789
G2	68	Serous papillary adenocarcinoma	T3cN0M0	48497910	38696105	12875871
G3	51	Serous papillary adenocarcinoma	T3cN1M0	21451250	4917044	2927058
G4	52	Serous adenocarcinoma	T3cN1M0	41594612	24305092	2332661
G5	51	Serous papillary adenocarcinoma	T3cN1M0	82060205	44946106	15094205
G6	54	Serous papillary adenocarcinoma	T3cN1M0	86714666	78252515	2824809
G7	66	Serous papillary adenocarcinoma	T3cN1M0	1.92E+08	1.58E+08	86760527
G8	26	Serous adenocarcinoma	T3cN1M0	2.03E+08	1.92E+08	1.84E+08
G9	41	Granulosa cell tumor	T2N0M0	1.02E+08	61551110	3655124
G10	68	Serous papillary adenocarcinoma	T3cN0M0	49955003	26374276	18547636
G11	51	Serous papillary adenocarcinoma	T3cN1M0	83494792	41128333	1752751
G13	51	Serous papillary adenocarcinoma	T3cN1M0	45191560	20807624	6173111
G14	54	Serous papillary adenocarcinoma	T3cN1M0	81667950	90127100	1917293

G15	66	Serous papillary adenocarcinoma	T3cN1M0	1.9E+08	85395127	2.55E+08
G16	26	Serous adenocarcinoma	T3cN1M0	82429177	9396364	1.87E+08
H1	38	Serous papillary adenocarcinoma	T3bN1M0	88166767	90634267	68743887
H2	48	Serous adenocarcinoma	T3cN0M0	70245846	63423850	3358198
H3	48	Serous papillary adenocarcinoma	T2N1M0	74096571	52978422	4385282
H4	57	Serous papillary adenocarcinoma	T3cN1M0	1.59E+08	85340333	87358568
H5	42	Serous adenocarcinoma	T1N1M0	1.76E+08	1.16E+08	1.6E+08
H6	64	Serous adenocarcinoma	T3cN1M0	1.51E+08	1.55E+08	50301620
H7	54	Serous adenocarcinoma	T2N1M0	97653074	50569155	52505296
H8	32	Serous adenocarcinoma	T3cN0M0	1.07E+08	38057254	63472167
H9	38	Serous papillary adenocarcinoma	T3bN1M0	87154842	51943262	1.35E+08
H10	48	Serous adenocarcinoma	T3cN0M0	49216996	35733994	5100034
H11	48	Serous papillary adenocarcinoma	T2N1M0	62017496	26158750	38690511
H12	57	Serous papillary adenocarcinoma	T3cN1M0	71601732	23611585	29108249
H13	42	Serous adenocarcinoma	T1N1M0	8625300	462422	28661321
H14	64	Serous adenocarcinoma	T3cN1M0	2.04E+08	1.71E+08	1.83E+08
H15	54	Serous adenocarcinoma	T2N1M0	1.05E+08	91400128	9621535
H16	32	Serous adenocarcinoma	T3cN0M0	85672805	51286241	11482580
I1	62	Serous adenocarcinoma	T3N0M0	57463400	45717932	4716196
I2	60	Serous adenocarcinoma	T3cN0M0	69042071	64482141	1133675
I3	66	Serous adenocarcinoma	T2N1M0	38392009	31665439	8167520
I4	56	Serous adenocarcinoma	T3N1M0	46390784	31681989	6915891
I5	46	Serous adenocarcinoma	T3aNIM0	1.8E+08	85354947	2.64E+08
I6	46	Serous adenocarcinoma	T2N1M0	1.45E+08	98300923	1.23E+08
I8	49	Serous adenocarcinoma	T3N1M0	92702618	53854715	15931219
I9	62	Serous adenocarcinoma	T3N0M0	64114263	50048092	1712080
I10	60	Serous adenocarcinoma	T3cN0M0	1.1E+08	85142305	1464084
I11	66	Serous adenocarcinoma	T2N1M0	41537082	19635958	1793884
I12	56	Serous adenocarcinoma	T3N1M0	45221056	31922619	8025159
I13	46	Serous adenocarcinoma	T3aNIM0	2.2E+08	1.46E+08	2.69E+08
I14	46	Serous adenocarcinoma	T2N1M0	1.55E+08	1.09E+08	1.13E+08
I15	47	Serous adenocarcinoma	T3cN1M0	96244345	1.07E+08	1225059
I16	49	Serous adenocarcinoma	T3N1M0	1.15E+08	1.05E+08	5875082
J1	57	Serous papillary adenocarcinoma	T3cN0M0	63943168	25323491	98209738
J2	46	Serous adenocarcinoma	T2cNIM0	1.09E+08	79542901	29129841
J3	57	Serous papillary adenocarcinoma	T3cN1M0	1.37E+08	56723093	69850520

J4	42	Serous papillary adenocarcinoma	T3cN1M0	76279921	38714715	35654079
J5	65	Serous papillary adenocarcinoma	T3cN0M0	1.02E+08	85442945	11563175
J6	66	Mucinous adenocarcinoma (mucago and blood vessel)	T3cN0M0	47355981	25322081	15277510
J7	67	Clear cell carcinoma	T3N2M0	46748724	63596277	37076070
J8	55	Transitional cell carcinoma	T3cN0M0	80739952	80526905	1402083
J9	57	Serous papillary adenocarcinoma	T3cN0M0	1.28E+08	55323336	90194594
J10	46	Serous adenocarcinoma	T2cNIM0	99661200	54051809	64127706
J11	57	Serous papillary adenocarcinoma	T3cN1M0	78096008	34103421	54037203
J12	42	Serous papillary adenocarcinoma	T3cN1M0	1.32E+08	98199782	90312302
J13	65	Serous papillary adenocarcinoma	T3cN0M0	1.17E+08	1.13E+08	3575409
J15	67	Clear cell carcinoma	T3N2M0	26519694	32710092	41799326
J16	55	Transitional cell carcinoma	T3cN0M0	45267433	59531673	1767077
K1	62	Malignant granulosa cell tumor	T2N0M0	1.13E+08	97000850	36723584
K2	38	Serous papillary adenocarcinoma (stroma)	-	93602035	60793075	46384177
K3	69	Serous papillary adenocarcinoma	-	1.27E+08	1.41E+08	1256875
K4	66	Serous papillary adenocarcinoma	-	1.04E+08	45890397	22136250
K5	65	Serous papillary adenocarcinoma	-	1.18E+08	1.04E+08	1560191
K6	37	Serous adenocarcinoma	-	98880812	69667971	38550545
K7	56	Serous papillary adenocarcinoma	-	47957235	58374700	5749585
K8	54	Serous papillary adenocarcinoma	-	50313240	51214755	1824937
K9	62	Malignant granulosa cell tumor	T2N0M0	1.23E+08	12353313	95813823
K10	38	Serous papillary adenocarcinoma	-	46941224	10814350	55030910
K11	69	Serous papillary adenocarcinoma	-	40993089	38608041	2193635
K12	66	Serous papillary adenocarcinoma	-	1.19E+08	46290130	41158050
K13	65	Serous papillary adenocarcinoma	-	62539933	42630967	10674467
K14	37	Serous adenocarcinoma	-	1.43E+08	46107163	2.15E+08

K15	56	Serous papillary adenocarcinoma	-	78444550	69825668	27203186
K16	54	Serous papillary adenocarcinoma	-	47074418	55675534	1771351
L1	55	Serous adenocarcinoma	-	25760073	29911561	731800
L2	41	Serous adenocarcinoma	-	60778530	60748782	1273583
L3	56	Serous adenocarcinoma	-	1.13E+08	43951145	68581132
L4	50	Mucinous adenocarcinoma	-	80398799	46818260	8613476
L5	51	Mucinous adenocarcinoma	-	38257676	5958955	41309290
L6	58	Serous papillary adenocarcinoma with clear cell carcinoma	-	21052067	29459471	4175218
L7	59	Endodermal sinus carcinoma	-	47760277	54076679	1553034
L8	16	Endodermal sinus carcinoma	-	22260271	12877206	32817840
L9	55	Serous adenocarcinoma	-	28369710	15610045	8427005
L10	41	Serous adenocarcinoma	-	29781530	22453782	10734708
L11	56	Serous adenocarcinoma	-	90185625	38897175	1.4E+08
L12	50	Mucinous adenocarcinoma	-	1.02E+08	44219152	53130918
L13	51	Mucinous adenocarcinoma	-	54604100	7216159	66854466
L14	58	Serous papillary adenocarcinoma with clear cell carcinoma	-	27049336	23928014	2413366
L15	59	Endodermal sinus carcinoma	-	52619346	60828208	518613
L16	16	Endodermal sinus carcinoma	-	30036751	8729810	26158221

Table S2. OV1501b TMA.

Position	Age	Pathology Diagnosis	TNM	Full RADD	oxRADD	PD-L1
A1	54	Ovary tissue	-	38189629	4080686	9940911
A2	53	Adjecent normal ovary tissue	-	69195228	31630619	1.79E+08
A3	48	Mucinous adenocarcinoma	T1aN0M0	61147450	22723130	8034127
A4	46	Theca cell tumor	-	57090519	45768812	50753206
A5	30	Borderline serous papillary cystadenoma	-	62707763	31992408	19385811
A6	47	High grade serous carcinoma	T3cN1M0	54774082	8916683	1449589
A7	49	High grade serous carcinoma	T2N0M0	56511506	2260742	60696269
A8	41	Metastatic adenocarcinoma	-	30445713	9660247	7464648

A9	25	Low grade serous carcinoma	T1N0M0	22638056	15154574	59219012
A10	55	Endometrioid adenocarcinoma	T1N0M0	59914211	1672523	4008831
A11	34	Low grade serous carcinoma	T1bN0M0	9395017	7599493	31041794
A12	41	Endometrioid adenocarcinoma	T1aN0M0	1.67E+08	41611754	3.2E+08
A13	49	Endometrioid adenocarcinoma	T1aN0M0	1.35E+08	8176664	1.08E+08
A14	34	Low grade serous carcinoma	T1aN0M0	3970939	1147104	3041548
A15	54	High grade serous carcinoma	T1aN0M0	92987764	5480120	2.79E+08
B1	54	Ovary tissue	-	37053711	8270548	10223759
B2	53	Adjecent normal ovary tissue	-	70184641	21623157	2.13E+08
B3	48	Mucinous adenocarcinoma	T1aN0M0	48639488	17349353	19525126
B4	46	Theca cell tumor	-	38564982	17042143	31837607
B5	30	Borderline serous papillary cystadenoma	-	44942164	9485527	48819879
B6	47	High grade serous carcinoma	T3cN1M0	43785565	17330775	3904577
B7	49	High grade serous carcinoma	T2N0M0	1.03E+08	33862954	2.12E+08
B8	41	Metastatic adenocarcinoma	-	80925089	70092840	4807915
B9	25	Low grade serous carcinoma	T1N0M0	1.09E+08	72883251	2.55E+08
B10	55	Endometrioid adenocarcinoma	T1N0M0	1.72E+08	1.35E+08	6417943
B11	34	Low grade serous carcinoma	T1bN0M0	70448365	51481175	43788357
B12	41	Endometrioid adenocarcinoma	T1aN0M0	2.3E+08	2.14E+08	2.17E+08
B13	49	Endometrioid adenocarcinoma	T1aN0M0	1.68E+08	1.21E+08	1.95E+08
B14	34	Low grade serous carcinoma	T1aN0M0	1.15E+08	99409186	14305611
B15	54	High grade serous carcinoma	T1aN0M0	1.12E+08	36758961	3.95E+08
C1	68	High grade serous carcinoma	T1aN0M0	1.11E+08	1.31E+08	36500805
C2	42	High grade serous carcinoma	T2N0M0	1.82E+08	2.27E+08	29062593

C3	26	Undifferentiated carcinoma	T1aN0M0	51643154	1.31E+08	7662460
C4	43	Endometrioid adenocarcinoma	T1aN0M0	87375850	1.27E+08	9768760
C5	46	High grade serous carcinoma	T2cN1M0	63872878	1.09E+08	30306205
C6	53	Endometrioid adenocarcinoma	T2aN0M0	1.2E+08	1.29E+08	4788524
C7	42	High grade serous carcinoma	T3cN1M0	1.81E+08	2.2E+08	23835744
C8	57	High grade serous carcinoma	T3cN1M0	1.46E+08	1.78E+08	21046378
C9	54	Endometrioid adenocarcinoma	T1bN0M0	1.22E+08	54337880	5287497
C10	42	Mucinous adenocarcinoma	T1aN0M0	1.09E+08	1.02E+08	28298428
C11	40	Mucinous adenocarcinoma	T1aN0M0	70596622	61795641	23976816
C12	61	Endometrioid adenocarcinoma	T3N0M0	1.12E+08	74244398	2.49E+08
C13	24	Undifferentiated carcinoma	T1aN0M0	1.7E+08	1.65E+08	16073444
C14	22	Low grade serous carcinoma	T2bN0M0	1.14E+08	1.25E+08	44987626
C15	48	High grade serous carcinoma	T1N0M0	94045564	75444679	4760914
D1	68	High grade serous carcinoma	T1aN0M0	1.42E+08	1.08E+08	90844971
D2	42	High grade serous carcinoma	T2N0M0	1.03E+08	1.42E+08	33219912
D3	26	Undifferentiated carcinoma	T1aN0M0	65863412	1.09E+08	11968683
D4	43	Endometrioid adenocarcinoma	T1aN0M0	87048070	1.16E+08	9480310
D5	46	High grade serous carcinoma	T2cN1M0	1.25E+08	1.44E+08	64483780
D6	53	Endometrioid adenocarcinoma	T2aN0M0	86151122	1.13E+08	2461169
D7	42	High grade serous carcinoma	T3cN1M0	1.52E+08	1.27E+08	1.95E+08
D8	57	High grade serous carcinoma	T3cN1M0	1.05E+08	1.18E+08	91962406
D9	54	Endometrioid adenocarcinoma	T1bN0M0	1.65E+08	1.09E+08	34080076
D10	42	Mucinous adenocarcinoma	T1aN0M0	60665873	27420650	29957069

D11	40	Mucinous adenocarcinoma	T1aN0M0	77427665	89431067	11801988
D12	61	Endometrioid adenocarcinoma	T3N0M0	1.72E+08	93925419	1.63E+08
D13	24	Undifferentiated carcinoma	T1aN0M0	1.97E+08	2.15E+08	18369286
D14	22	Low grade serous carcinoma	T2bN0M0	99983159	1.03E+08	21995407
D15	48	High grade serous carcinoma	T1N0M0	1.26E+08	1.63E+08	17226091
E1	54	Mucinous adenocarcinoma	-	37922535	7221626	36289527
E2	60	Endometrioid adenocarcinoma	T1bN0M0	1.39E+08	1.2E+08	86470068
E3	44	Endometrioid adenocarcinoma	T1N0M0	1.78E+08	2.13E+08	3868292
E4	44	Endometrioid adenocarcinoma	T1aN0M0	99301650	1.1E+08	23407356
E5	48	Mucinous adenocarcinoma	T1bN0M0	32284631	32572333	1067046
E6	52	Endometrioid adenocarcinoma	T1aN0M0	1.59E+08	1.63E+08	11653720
E7	16	Endodermal sinus carcinoma	T2N0M0	84827966	1.17E+08	20004160
E8	22	Endodermal sinus carcinoma	T1cN0M0	57033994	73920490	15673715
E9	38	Low grade serous carcinoma	T3cN1M0	52257696	40710749	56536370
E10	59	Endometrioid adenocarcinoma	T2bN0M0	1.3E+08	1.57E+08	7354473
E11	46	Endometrioid adenocarcinoma	T2N0M0	67094479	26526505	6769506
E12	54	Endometrioid adenocarcinoma	T1cN0M0	1.22E+08	1.41E+08	5570482
E13	47	Endometrioid adenocarcinoma	T2aN0M0	65281202	65167217	26901757
E14	42	Mucinous adenocarcinoma	T1aN0M0	92380554	86530535	8432604
E15	29	Mucinous adenocarcinoma	T3N0M0	1.91E+08	1.4E+08	54656972
F1	54	Mucinous adenocarcinoma	-	48401867	48401867	17006208
F2	60	Endometrioid adenocarcinoma	T1bN0M0	1.78E+08	1.78E+08	34631264
F3	44	Endometrioid adenocarcinoma	T1N0M0	1.5E+08	1.5E+08	18936305

F4	44	Endometrioid adenocarcinoma	T1aN0M0	82259045	82259045	11086924
F5	48	Mucinous adenocarcinoma	T1bN0M0	65230638	65230638	2360817
F6	52	Endometrioid adenocarcinoma	T1aN0M0	1.5E+08	1.5E+08	16633375
F7	16	Endodermal sinus carcinoma	T2N0M0	1.15E+08	1.15E+08	26905808
F8	22	Endodermal sinus carcinoma	T1cN0M0	75305212	75305212	50270261
F9	38	Low grade serous carcinoma	T3cN1M0	90496578	90496578	81880575
F10	59	Endometrioid adenocarcinoma	T2bN0M0	1.54E+08	1.54E+08	5299689
F11	46	Endometrioid adenocarcinoma	T2N0M0	69915285	69915285	12019488
F12	54	Endometrioid adenocarcinoma	T1cN0M0	1.12E+08	1.12E+08	4522816
F13	47	Endometrioid adenocarcinoma	T2aN0M0	58282477	58282477	2644924
F14	42	Mucinous adenocarcinoma	T1aN0M0	1.03E+08	1.03E+08	3989418
F15	29	Mucinous adenocarcinoma	T3N0M0	1.83E+08	1.83E+08	43402933
G1	45	High grade serous carcinoma	T1aN0M0	77888789	19403347	1.76E+08
G2	46	Lowly malignant fibrosarcoma	T2N0M0	43491704	94226988	2703940
G3	55	High grade serous carcinoma	T1N0M0	1.79E+08	1.71E+08	2.07E+08
G4	58	High grade serous carcinoma	T1N0M0	1.45E+08	1.46E+08	1.13E+08
G5	32	Endodermal sinus carcinoma	T2bN0M0	1.27E+08	1.58E+08	5446823
G6	47	High grade serous carcinoma	T1aN0M0	2.25E+08	2.35E+08	1.96E+08
G7	60	Mucinous adenocarcinoma	T1aN0M0	8014733	7921094	5467906
G8	52	High grade serous carcinoma	T3N0M0	1.77E+08	1.74E+08	2.07E+08
G9	47	Endometrioid adenocarcinoma	T2aN0M0	1.94E+08	2.2E+08	4981289
G10	34	Endometrioid adenocarcinoma	T1N0M0	1.47E+08	1.26E+08	48228737
G11	19	Mucinous adenocarcinoma	T2aN0M0	84481256	88575899	5396019
G12	44	Granular cell tumor	T1cN0M0	2.58E+08	2.21E+08	1.66E+08

G13	65	High grade serous carcinoma	T2N0M0	1.8E+08	1.26E+08	1.86E+08
G14	47	Endometrioid adenocarcinoma	T1cN0M0	1.4E+08	1.21E+08	48654694
G15	68	Endometrioid adenocarcinoma	T2cN0M0	1.23E+08	81889929	11136307
H1	45	High grade serous carcinoma	T1aN0M0	98708300	14428187	2.6E+08
H2	46	Lowly malignant fibrosarcoma	T2N0M0	35740756	71811378	5497185
H3	55	High grade serous carcinoma	T1N0M0	1.5E+08	1.3E+08	2.05E+08
H4	58	High grade serous carcinoma	T1N0M0	2.02E+08	2.49E+08	94419864
H5	32	Endodermal sinus carcinoma	T2bN0M0	90454363	1.72E+08	9569600
H6	47	High grade serous carcinoma	T1aN0M0	2.33E+08	2.27E+08	1.13E+08
H7	60	Mucinous adenocarcinoma	T1aN0M0	4314876	4436895	4348589
H8	52	High grade serous carcinoma	T3N0M0	88615455	1.17E+08	37227512
H9	47	Endometrioid adenocarcinoma	T2aN0M0	91466716	1.78E+08	3936449
H10	34	Endometrioid adenocarcinoma	T1N0M0	1.14E+08	1.59E+08	44228776
H11	19	Mucinous adenocarcinoma	T2aN0M0	30590028	31955347	5279454
H12	44	Granular cell tumor	T1cN0M0	2.41E+08	2.27E+08	1.97E+08
H13	65	High grade serous carcinoma	T2N0M0	1.67E+08	1.31E+08	2.25E+08
H14	47	Endometrioid adenocarcinoma	T1cN0M0	69684525	73049409	49826598
H15	68	Endometrioid adenocarcinoma	T2cN0M0	58277424	45742335	10512635
I1	12	Dysgerminoma	T1bN0M0	1.04E+08	1344747	2.52E+08
I2	55	Endometrioid adenocarcinoma	T1cN0M0	45184525	55398519	6413749
I3	44	High grade serous carcinoma	T1aN0M0	1.64E+08	15667587	5.68E+08
I4	52	High grade serous carcinoma	T1aN0M0	89201740	11580470	1.92E+08
I5	61	Mucinous carcinoma	T1cN0M0	53526865	59784481	3498453
I6	65	Endometrioid adenocarcinoma	T1aN0M0	1.3E+08	1.91E+08	95410554

I7	40	Mucinous adenocarcinoma	T1cN0M0	15137528	20512696	2462519
I8	50	Endometrioid adenocarcinoma	T1aN0M0	1.32E+08	1.11E+08	1.1E+08
I9	40	High grade serous carcinoma	T1aN0M0	1.1E+08	1.03E+08	1.15E+08
I10	43	Endometrioid adenocarcinoma	T1cN0M0	49284687	5317113	53619682
I11	54	High grade serous carcinoma	T3cN0M0	89020171	1.82E+08	13128758
I12	47	Endometrioid adenocarcinoma	T1cN0M0	26953465	31721948	21188719
I13	55	High grade serous carcinoma	T1cN0M0	1.69E+08	2.05E+08	2.45E+08
I14	34	Mucinous adenocarcinoma	T1aN0M0	54538618	58091463	84638450
I15	56	Mucinous adenocarcinoma	T1N0M0	10910047	15594096	4824936
J1	12	Dysgerminoma	T1bN0M0	65986436	116013	2.24E+08
J2	55	Endometrioid adenocarcinoma	T1cN0M0	22373965	17328330	8359661
J3	44	High grade serous carcinoma	T1aN0M0	1.14E+08	1244644	3.6E+08
J4	52	High grade serous carcinoma	T1aN0M0	53440004	1574737	75740651
J5	61	Mucinous carcinoma	T1cN0M0	24286700	47370131	989900
J6	65	Endometrioid adenocarcinoma	T1aN0M0	52479139	82467714	63879752
J7	40	Mucinous adenocarcinoma	T1cN0M0	6070642	6630725	1769486
J8	50	Endometrioid adenocarcinoma	T1aN0M0	1.82E+08	37011990	3.97E+08
J9	40	High grade serous carcinoma	T1aN0M0	51460908	40759650	35856962
J10	43	Endometrioid adenocarcinoma	T1cN0M0	31897326	3795581	61280504
J11	54	High grade serous carcinoma	T3cN0M0	3013899	2765771	1277535
J12	47	Endometrioid adenocarcinoma	T1cN0M0	15766833	21339066	6973290
J13	55	High grade serous carcinoma	T1cN0M0	36952261	49755455	45874657
J14	34	Mucinous adenocarcinoma	T1aN0M0	23880034	21810362	33798594
J15	56	Mucinous adenocarcinoma	T1N0M0	2497840	3222704	3180510

Table S3. BC110118A1 TMA.

Position	Age	Pathology Diagnosis	TNM	Full RADD	oxRADD	PD-L1
A1	53	High grade serous carcinoma	T2N0M1	15891942	106545	1695665
A2	42	High grade serous carcinoma	T1N0M0	51273969	34847687	10686068
A3	26	Low grade serous carcinoma	T1cN0M0	41914645	9459747	23385415
A4	51	High grade serous carcinoma	T3cN1M0	19723672	473264	2751123
A5	69	Mucinous adenocarcinoma	T1aN0M0	13660855	71699	17813869
A6	48	High grade serous carcinoma	T1N0M0	7702451	191460	3205776
A7	57	High grade serous carcinoma	T1cN0M0	62047296	2696294	2.44E+08
A8	50	High grade serous carcinoma	T2N0M0	38179084	8583684	97568468
A9	47	High grade serous carcinoma	T1N0M0	20426754	2109507	33164599
B1	56	High grade serous carcinoma	T2N0M0	56401186	29216186	4566151
B2	35	High grade serous carcinoma	T1aN0M0	1.46E+08	1.07E+08	62523382
B3	26	High grade serous carcinoma	T3cN1M0	1.87E+08	1.42E+08	1.74E+08
B4	22	High grade serous carcinoma	T2bN0M0	1.51E+08	26831942	1.18E+08
B5	53	High grade serous carcinoma	T1cN0M0	1.16E+08	12736377	65944287
B6	32	High grade serous carcinoma	T1N0M0	84107803	32873927	78970770
B7	46	High grade serous carcinoma	T1aN0M0	75299468	18638847	88640636
B8	50	High grade serous carcinoma	T1aN0M0	1.65E+08	32822786	21753598
B9	49	High grade serous carcinoma	T1aN0M0	1.05E+08	51966713	7336006
C1	52	Mucinous adenocarcinoma	T1N0M0	62097839	15968707	30528186
C2	38	High grade serous carcinoma	T3bN1M0	1.44E+08	1.4E+08	26594907
C3	49	High grade serous carcinoma	T2N0M0	2.27E+08	79941739	1.9E+08
C4	58	High grade serous carcinoma	T1N0M0	1.18E+08	16773335	1.57E+08

C5	50	High grade serous carcinoma	T1N0M0	1.11E+08	20491881	1.15E+08
C6	52	High grade serous carcinoma	T2aN0M0	53300664	2113514	1193901
C7	65	High grade serous carcinoma	T2N0M0	2.92E+08	31986122	1.51E+08
C8	46	High grade serous carcinoma	T2cN1M0	1.62E+08	41842878	1.08E+08
C9	54	High grade serous carcinoma	T3cN0M0	1.76E+08	89142707	55655481
D1	55	High grade serous carcinoma	T2N0M0	74314648	35659841	34098331
D2	42	High grade serous carcinoma	T3cN1M0	1.2E+08	10482092	1.03E+08
D3	47	High grade serous carcinoma	T1cN0M0	84585485	19763847	59027047
D4	51	High grade serous carcinoma	T1N0M0	39056450	12469054	8330356
D5	57	High grade serous carcinoma	T3cN1M0	1.4E+08	31713413	1.25E+08
D6	46	High grade serous carcinoma	T3aN1M0	2.51E+08	72188914	3.98E+08
D7	42	High grade serous carcinoma	T2N0M0	1.41E+08	8544728	56342521
D8	49	High grade serous carcinoma	T2N0M0	2.27E+08	40210318	72624214
D9	52	High grade serous carcinoma	T2N0M0	2.06E+08	1.26E+08	1.24E+08
E1	49	High grade serous carcinoma	T1N0M0	1.49E+08	1.45E+08	2.07E+08
E2	52	High grade serous carcinoma	T1aN0M0	90997067	47539623	1.62E+08
E3	49	High grade serous carcinoma	T1N0M0	1.2E+08	48009206	1.52E+08
E4	39	Endometrioid adenocarcinoma	T1aN0M0	53491570	39968858	80822002
E5	55	Endometrioid adenocarcinoma	T1N0M0	98816687	25899711	1.18E+08
E6	48	Clear-cell carcinoma	T2N0M0	1.05E+08	4127893	65338663
E7	43	Clear-cell carcinoma	T1N0M0	53413796	28780784	7281253
E8	40	Clear-cell carcinoma	T1N0M0	1.08E+08	52733190	30152321
E9	27	Endodermal sinus carcinoma	T1N0M0	74096861	951934	17603528
F1	44	Granular cell tumor	T1cN0M0	91039929	67979979	2.1E+08
F2	57	Granular cell tumor	T1N0M0	1.14E+08	25794655	71202714
F3	43	Granular cell tumor	T1N0M0	50032063	1179162	56345739

F4	13	Dysgerminoma	T1aN0M0	74562962	59905312	35361484
F5	35	Dysgerminoma	T1aN0M0	77862131	51224583	1.18E+08
F6	41	Dysgerminoma	T1N0M0	56900862	20406104	9477681
F7	34	Low grade serous carcinoma	T1aN0M0	19051716	12100124	68276747
F8	48	Malignant theca folliculi tumor	T3N0M0	1.83E+08	1.2E+08	2.34E+08
F9	24	Endodermal sinus carcinoma	T2N0M0	76785955	33470026	1.35E+08
G1	48	Metastatic adenocarcinoma	-	33036745	15798361	75809445
G2	40	Metastatic adenocarcinoma	-	7920619	1476305	23728773
G3	38	Metastatic adenocarcinoma	-	45908959	4400885	1.24E+08
G4	55	Metastatic adenocarcinoma	-	56573181	5785045	4.02E+08
G5	66	Metastatic adenocarcinoma	-	63592848	32741670	1.46E+08
G6	30	Metastatic adenocarcinoma	-	31476216	32868608	709211
G7	53	Metastatic adenocarcinoma	-	66046967	12302502	34439353
G8	31	Metastatic adenocarcinoma	-	80503988	45028955	1.37E+08
G9	62	Malignant change of benign teratoma (squamous cell carcinoma II)	-	78017604	15885543	57356202
H1	39	Adjacent normal ovary tissue	-	3429112	8263822	3533690
H2	49	Adjacent normal ovary tissue	-	27115232	8577707	1.09E+08
H3	48	Adjacent normal ovary tissue	-	9216120	854294	41340966
H4	42	Adjacent normal ovary tissue	-	24175896	31645929	25470925
H5	41	Adjacent normal ovary tissue	-	28519514	9020681	1.1E+08
H6	53	Adjacent normal ovary tissue	-	1.08E+08	28097016	74825052
H7	20	Ovary tissue	-	1.16E+08	58538805	5204113
H8	15	Ovary tissue	-	53779029	19825803	19973217
H9	19	Ovary tissue	-	22686297	3484556	376305
