# Supplementary figures and images for: Associations between DNA Damage and PD-L1 Expression in Ovarian Cancer, a Potential Biomarker for Clinical Response
Source: Biology (Basel). 2021 Apr 29;10(5):385. doi: 10.3390/biology10050385 (PMC8146974; doi:10.3390/biology10050385)

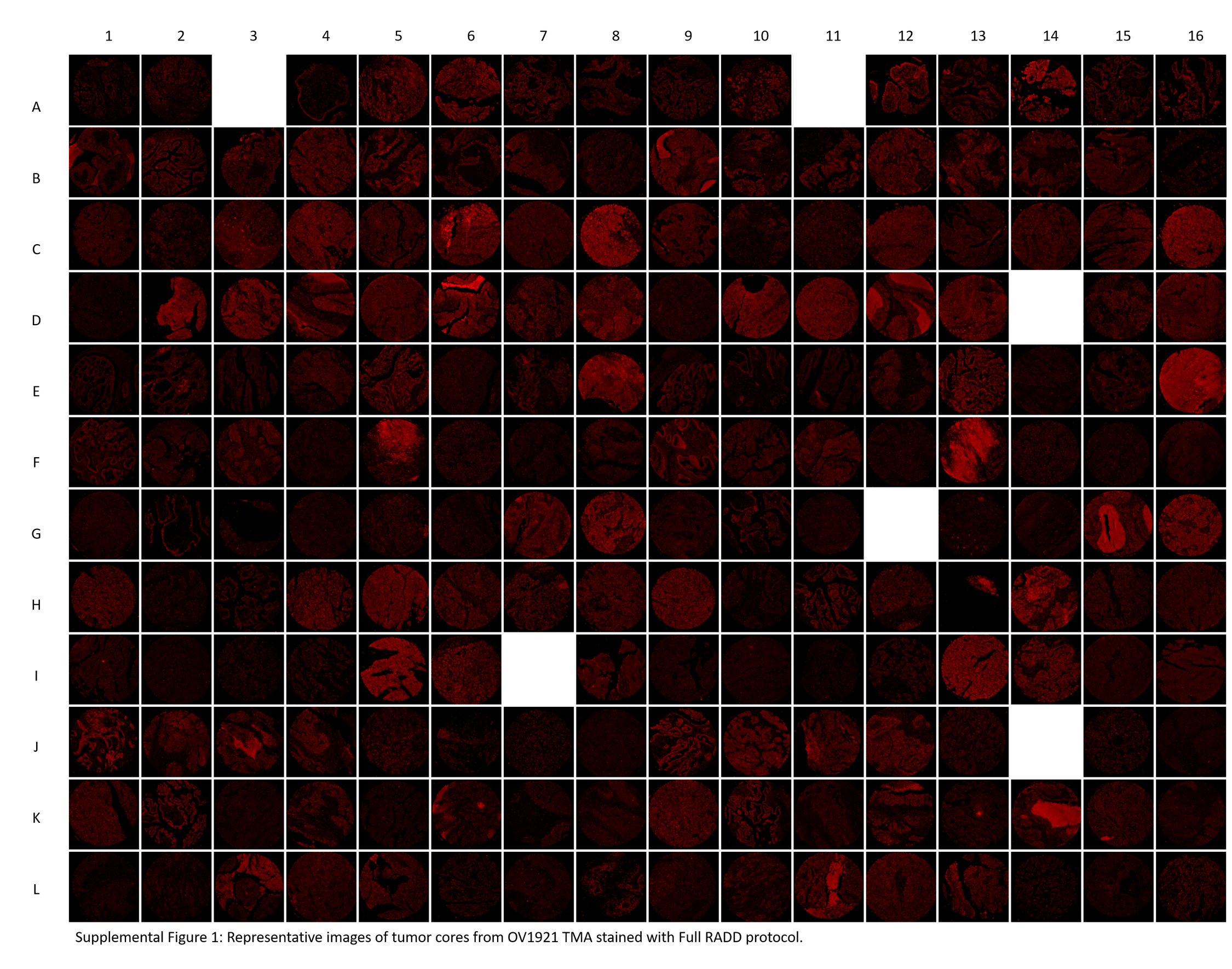

Supplement: Supplementary file 1 [file biology-10-00385-s001.zip › SupplementalFigure1.tif]

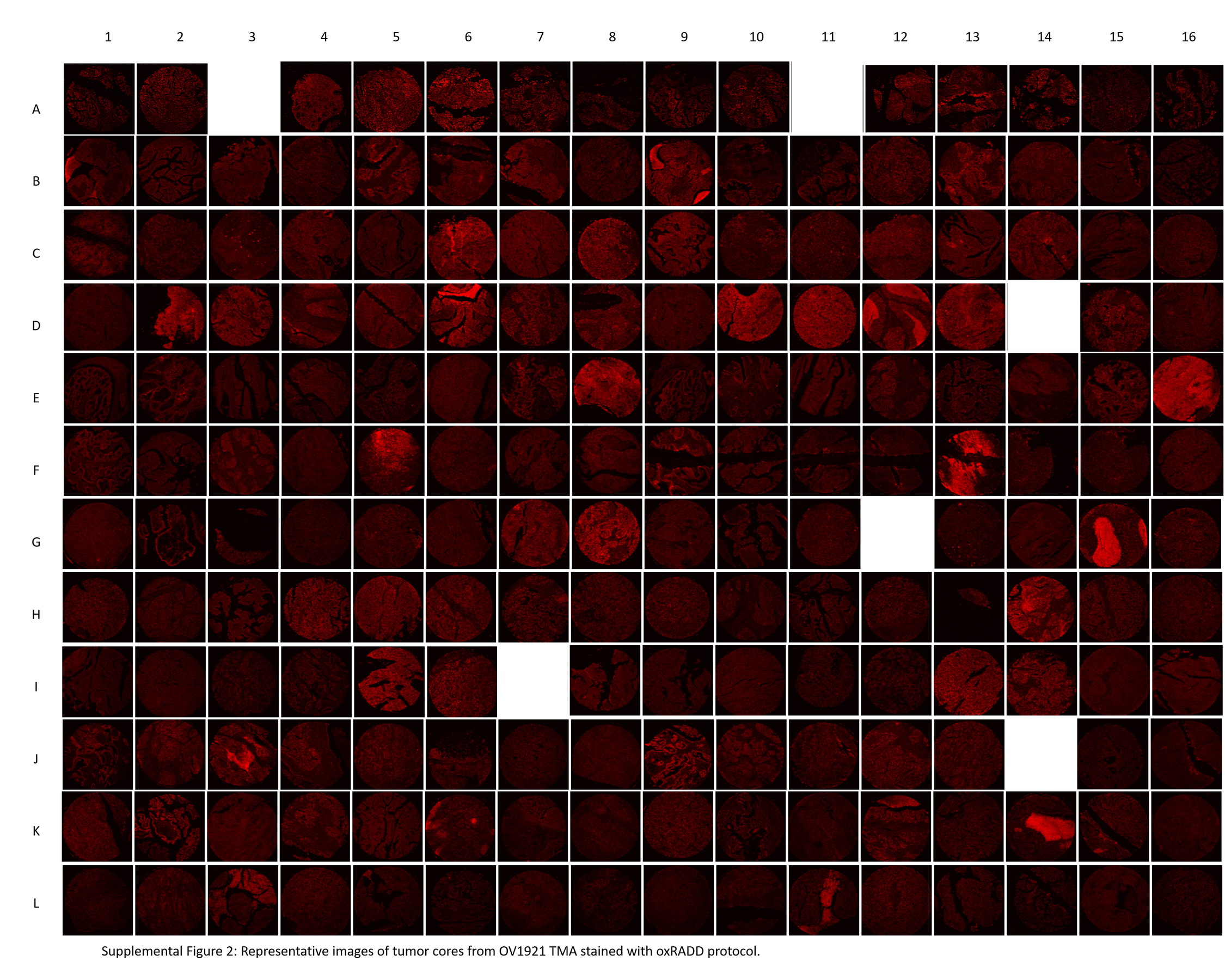

Supplement: Supplementary file 1 [file biology-10-00385-s001.zip › SupplementalFigure2.tif]

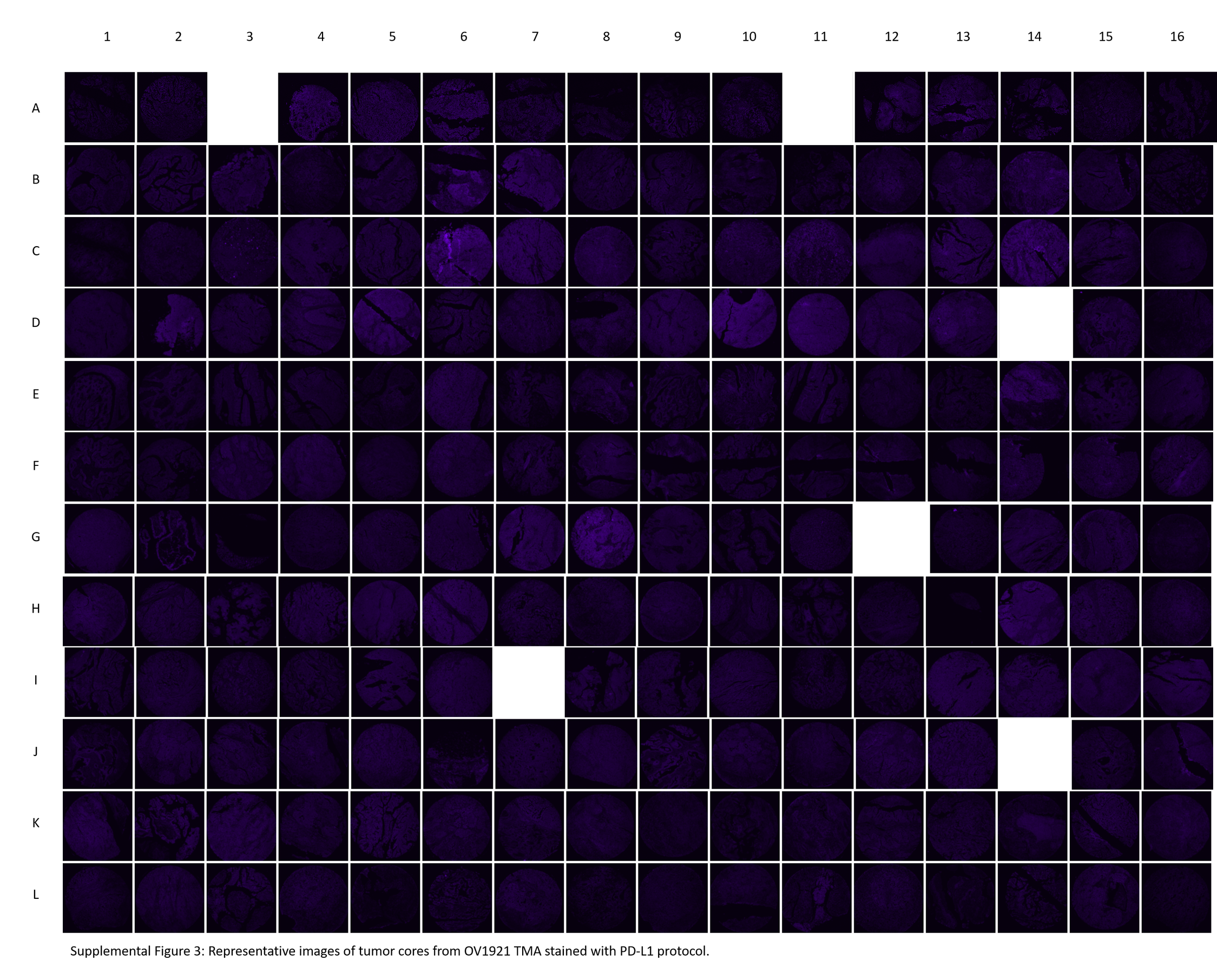

Supplement: Supplementary file 1 [file biology-10-00385-s001.zip › SupplementalFigure3.tif]

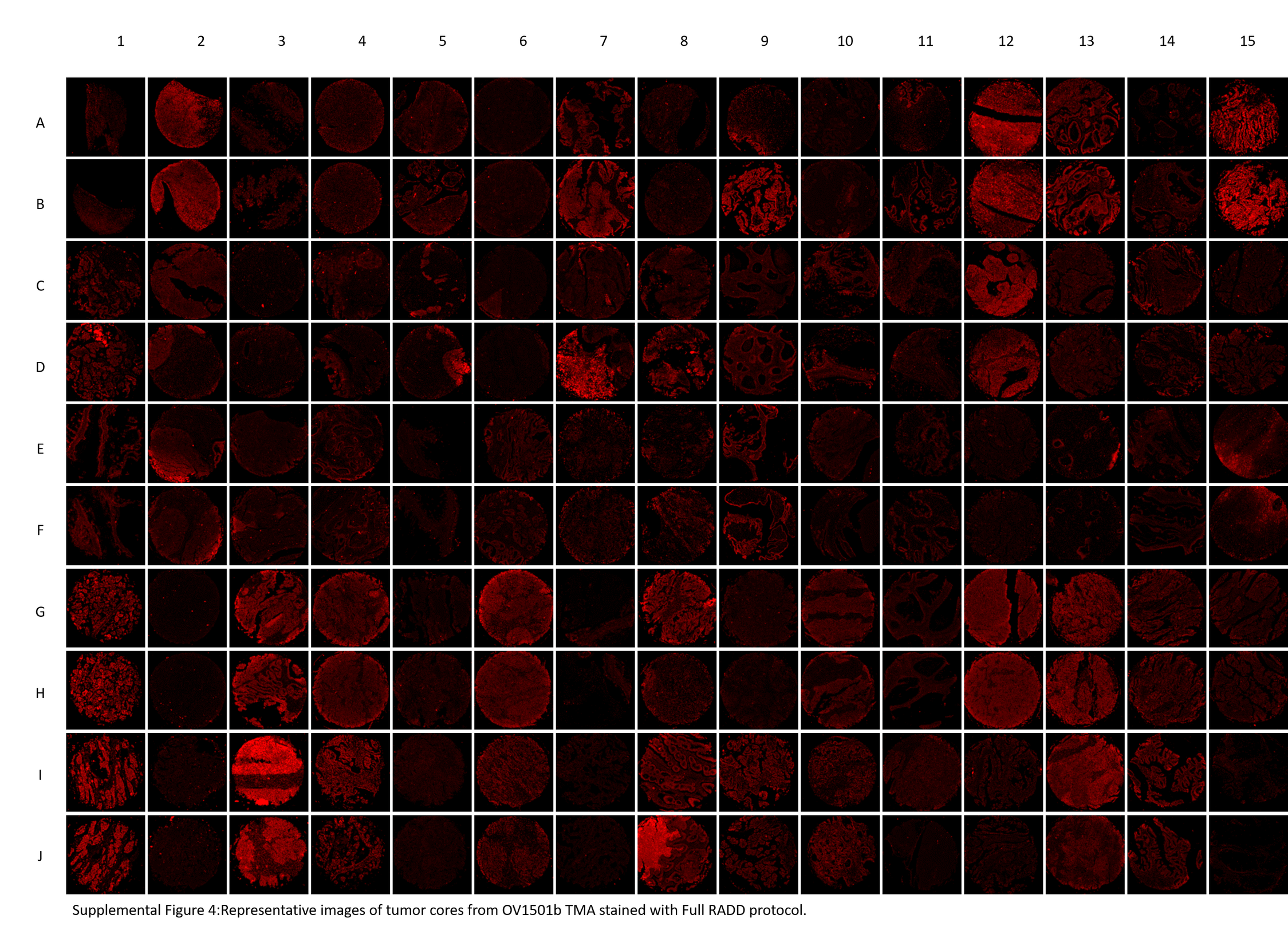

Supplement: Supplementary file 1 [file biology-10-00385-s001.zip › SupplementalFigure4.tif]

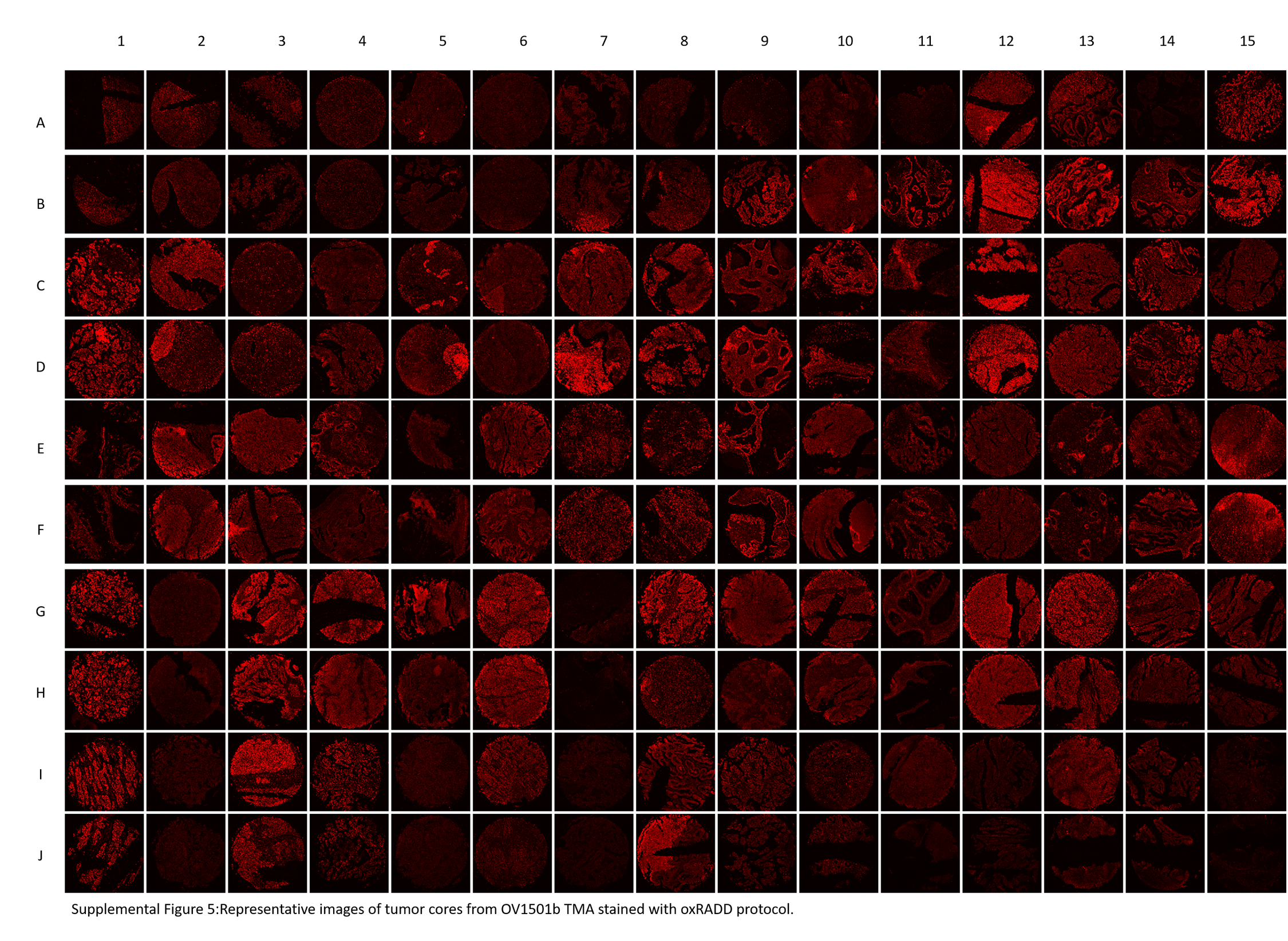

Supplement: Supplementary file 1 [file biology-10-00385-s001.zip › SupplementalFigure5.tif]

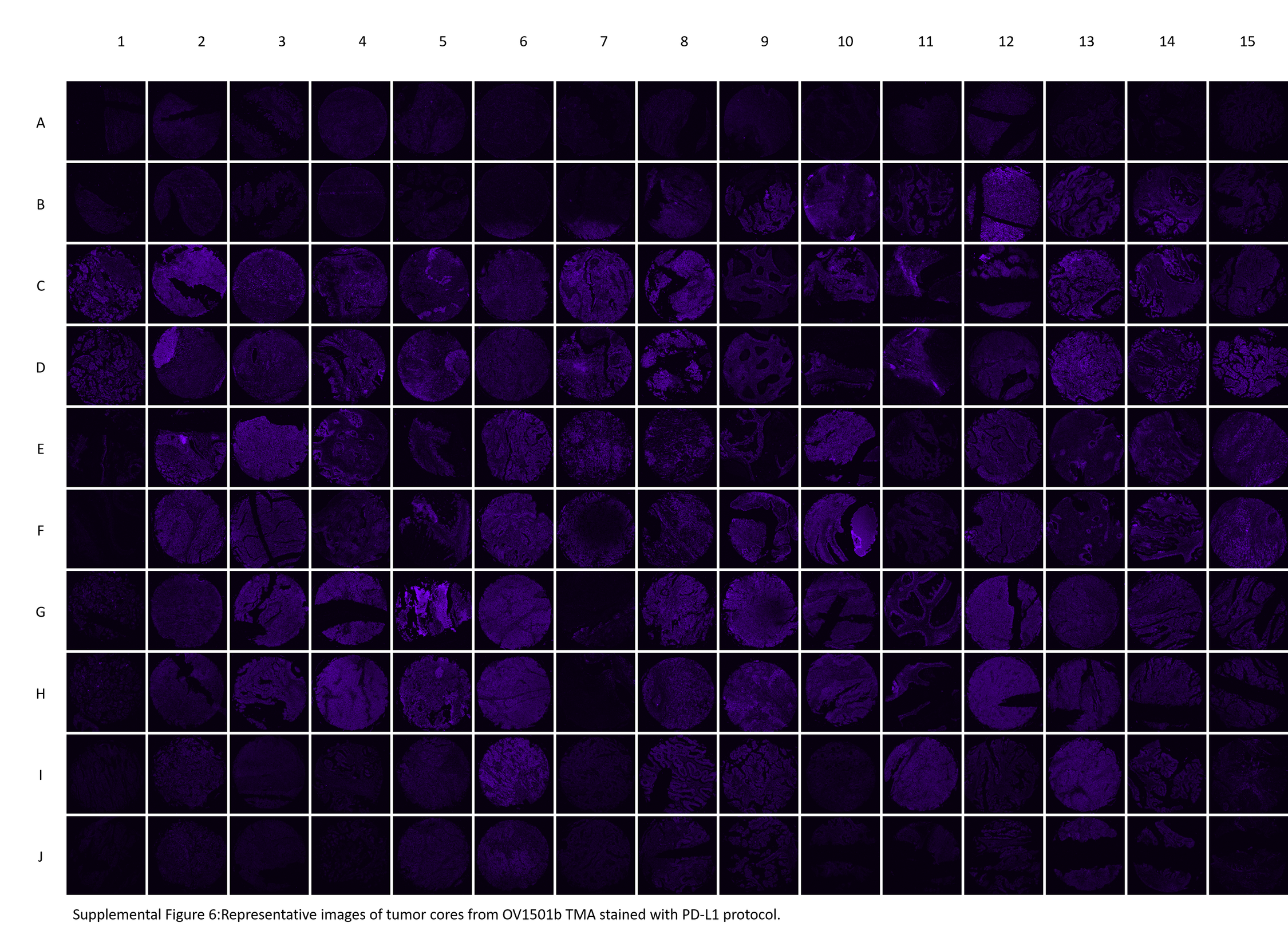

Supplement: Supplementary file 1 [file biology-10-00385-s001.zip › SupplementalFigure6.tif]

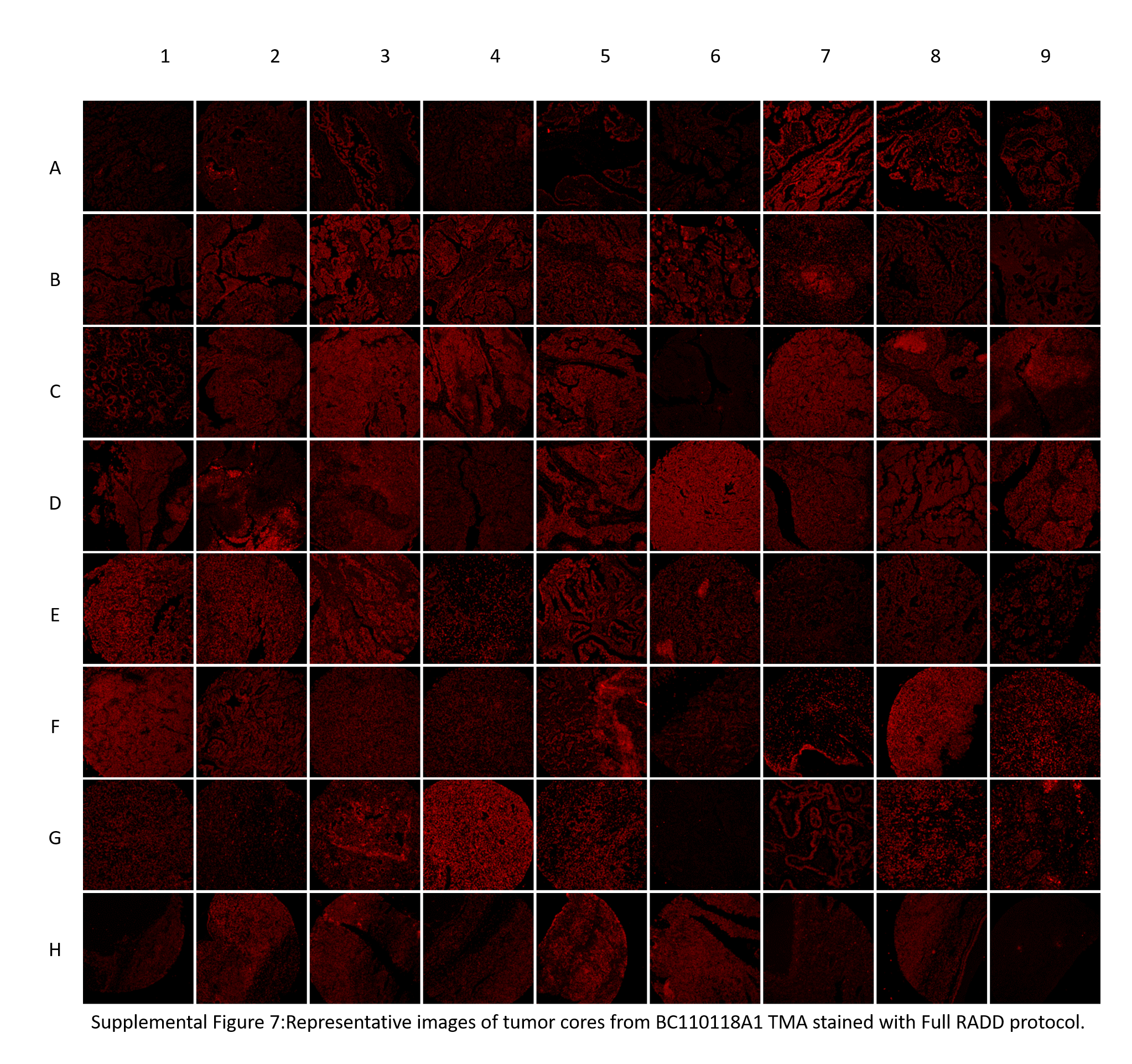

Supplement: Supplementary file 1 [file biology-10-00385-s001.zip › SupplementalFigure7.tif]

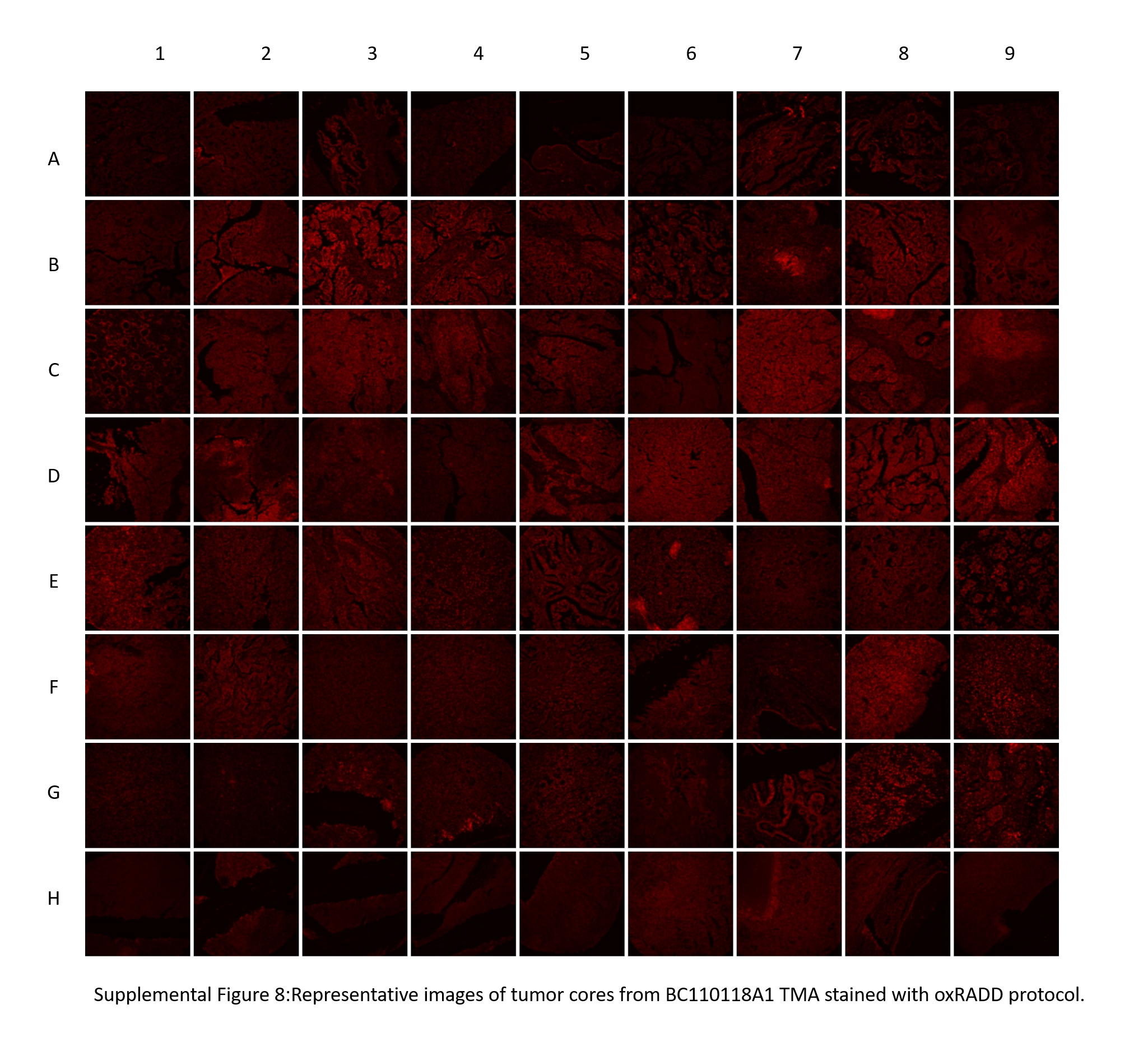

Supplement: Supplementary file 1 [file biology-10-00385-s001.zip › SupplementalFigure8.tif]

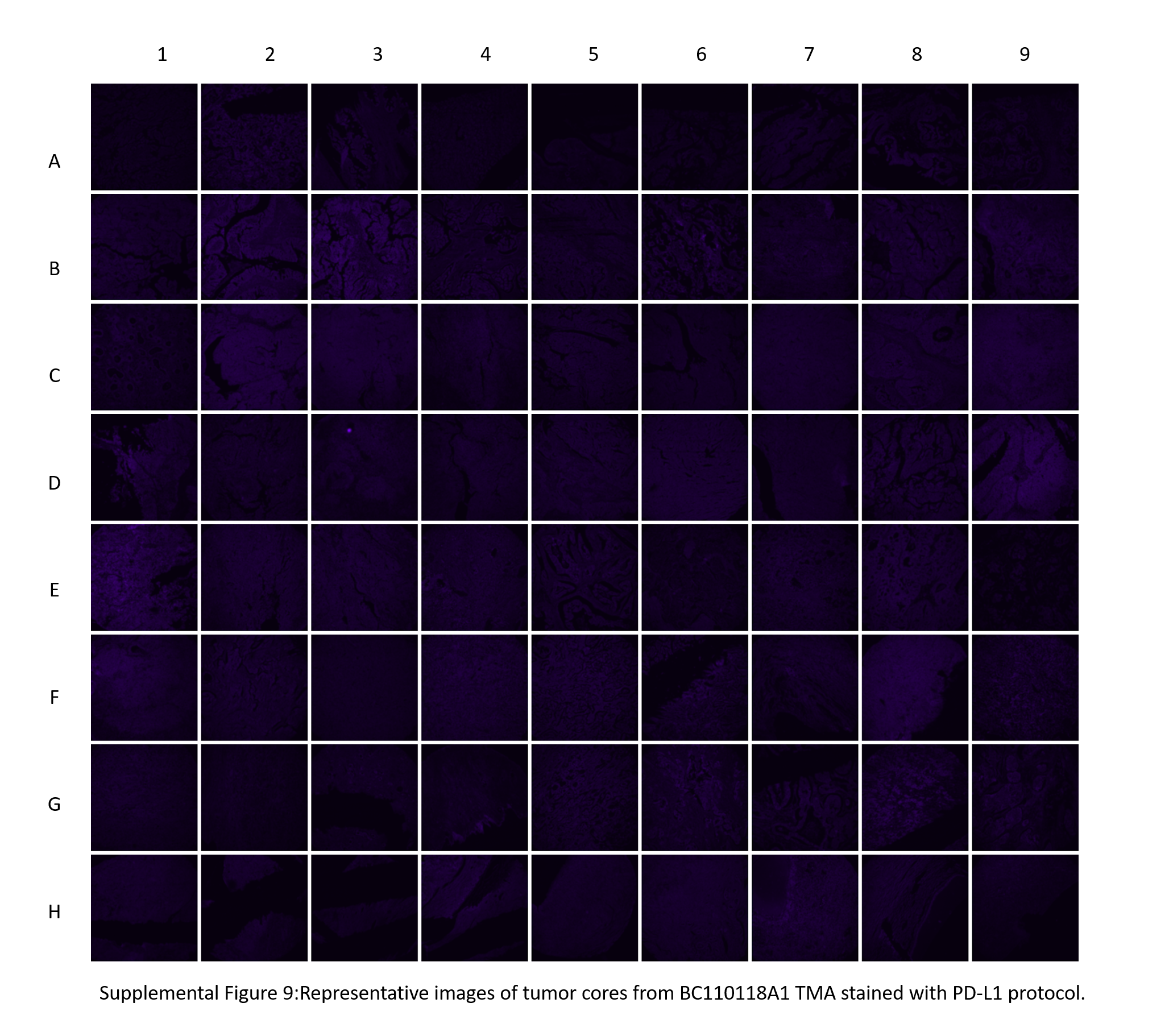

Supplement: Supplementary file 1 [file biology-10-00385-s001.zip › SupplementalFigure9.tif]
